# 4‐Hydroxytamoxifen induces a noncanonical ferroptosis in lung adenocarcinoma by targeting AKR1B10

**DOI:** 10.1002/ctm2.70796

**Published:** 2026-09-08

**Authors:** Hui Yang, Yanan Wang, Jiangbo Jin, Hanyan Xu, Yinshui Miao, Xiaojing Wu, Shengsong Chen, Tianyu Han, Qingyuan Zhan

**Affiliations:** ^1^ Jiangxi Provincial Key Laboratory of Respiratory Diseases Jiangxi Institute of Respiratory Diseases Jiangxi Clinical Research Center for Respiratory Diseases Department of Pulmonary and Critical Care Medicine The First Affiliated Hospital Jiangxi Medical College Nanchang University Nanchang Jiangxi Province P.R. China; ^2^ China–Japan Friendship Jiangxi Hospital National Regional Center for Respiratory Medicine Nanchang Jiangxi Province P.R. China; ^3^ Jiangxi Provincial Key Laboratory of Respiratory Diseases Jiangxi Institute of Respiratory Diseases Jiangxi Clinical Research Center for Respiratory Diseases Department of Thoracic Surgery The First Affiliated Hospital Jiangxi Medical College Nanchang University Nanchang Jiangxi Province P.R. China; ^4^ Department of Pulmonary and Critical Care Medicine Center of Respiratory Medicine China–Japan Friendship Hospital Beijing P.R. China

**Keywords:** AKR1B10, 4‐hydroxytamoxifen, lipid deficiency, lipid peroxidation, LUAD, noncanonical ferroptosis

## Abstract

**Background:**

Lung adenocarcinoma (LUAD) remains a leading cause of cancer mortality, and current therapies are limited by drug resistance and toxicity. Ferroptosis offers an attractive strategy for cancer therapy, but classical ferroptosis is iron‑dependent. Repurposing approved drugs offers a rapid strategy to identify novel anti‑LUAD agents, yet the mechanism by which 4‑Hydroxytamoxifen (4‑OHT) exerts estrogen receptor–independent anti‑tumour effects remains unclear.

**Methods:**

We performed a drug repurposing screen of 950 endogenous metabolites in LUAD cells.

Anti‑tumour activity was validated in vitro and in vivo using LUAD cell lines and mouse models. Target identification and mechanistic studies employed LiP‑MS, pull‐down SPR, CETSA, metabolomics, and enzyme activity assays. Clinical relevance was assessed via tissue microarrays and TCGA analysis. Drug synergy was quantified using the Chou–Talalay method.

**Results:**

4‑OHT inhibited LUAD cell proliferation with IC_50_ values of 3.86 ± 0.26 µM to 13.58 ± 0.55 µM independent of estrogen receptor. It triggered noncanonical ferroptosis characterised by GSH depletion (reduced by about 60%), GPX4 downregulation, lipid peroxidation (about 2.5‐fold increase), and iron independence. Mechanistically, 4‑OHT directly bound AKR1B10 (K_D_(M) was 7.98e‐06) and inhibited its enzymatic activity, leading to ACC1 degradation, lipid deficiency, and accumulation of toxic lipid‑derived carbonyls. AKR1B10 knockout abolished 4‑OHT‑induced cell death, while rescue restored sensitivity. Nanatinostat similarly targeted AKR1B10 and induced the same cell death modality. AKR1B10 upregulation in LUAD tissues correlated with reduced overall survival rates.

**Conclusion:**

This study demonstrates that 4‑OHT directly inhibits AKR1B10 enzymatic activity to induce a noncanonical ferroptosis in LUAD. AKR1B10 overexpression correlates with poor prognosis and chemoresistance in LUAD patients. Nonetheless, AKR1B10 inhibition warrants further investigation as a therapeutic strategy.

**Highlights:**

1. 4‑OHT directly inhibits AKR1B10 enzymatic activity to induce noncanonical ferroptosis in LUAD.
2. AKR1B10 sustains LUAD survival by stabilising ACC1 and detoxifying reactive carbonyl species (RCSs).
3. Nanatinostat similarly targets AKR1B10 and elicits the same cell death modality.
4. AKR1B10 overexpression correlates with poor prognosis and chemoresistance in LUAD.

## Graphical abstract

- 4‑OHT directly inhibits AKR1B10 enzymatic activity to induce noncanonical ferroptosis in LUAD.
- AKR1B10 sustains LUAD survival by stabilising ACC1 and detoxifying reactive carbonyl species (RCSs).
- Nanatinostat similarly targets AKR1B10 and elicits the same cell death modality.
- AKR1B10 overexpression correlates with poor prognosis and chemoresistance in LUAD.

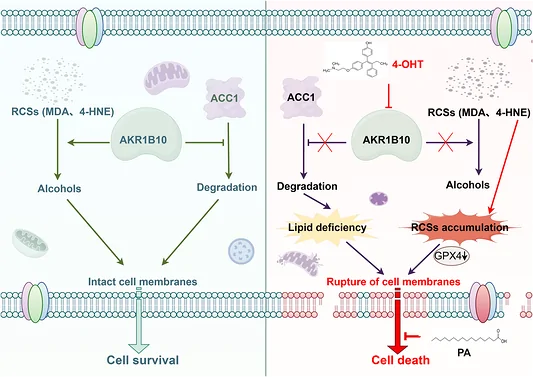

## 1 INTRODUCTION

Lung adenocarcinoma (LUAD) represents a major histological subtype of non‐small cell lung cancer (NSCLC) and poses a substantial global health burden, with rising incidence and persistently high mortality rates worldwide.^1^, ^2^ The current therapeutic strategies for LUAD often face formidable challenges such as drug resistance, recurrence, and severe side effects, which limit the overall efficacy and patient survival.^3^, ^4^ Therefore, new therapeutic targets and strategies are urgently needed to improve LUAD patient outcomes.

Cell death is fundamental to tissue homeostasis and the removal of abnormal cells, including cancer cells.^5^ Classical forms of programmed cell death include apoptosis, necroptosis, autophagic cell death, and ferroptosis.^6^, ^7^, ^8^, ^9^, ^10^ Among these, classical ferroptosis is triggered by System Xc^−^ inhibition, glutathione (GSH) depletion, and GPX4 inactivation, which collectively result in iron‐catalysed lipid peroxidation.^10^, ^11^ However, accumulating evidence indicates that noncanonical ferroptosis encompasses cell death pathways that operate independently of GPX4 or are triggered by stimuli distinct from classical inducers (e.g., erastin/RSL3). These pathways involve unique effectors and regulatory axes, including the FSP1‑CoQ10‑NAD(P)H system that operates independently of GPX4 and the recently identified GPX1‐OSBPL8 axis, which suppresses endoplasmic reticulum‑localised phosphatidic acid peroxidation through GPX1‑mediated reduction of oxidised PA.^12^, ^13^ Collectively, these findings highlight the mechanistic and spatial heterogeneity of ferroptosis beyond the canonical System Xc^−^–GPX4 paradigm. Importantly, noncanonical ferroptosis has shown considerable potential in suppressing tumour proliferation in preclinical models, highlighting its therapeutic relevance and promising clinical translatability.^14^, ^15^, ^16^, ^17^

Drug repurposing refers to the process of developing new medical applications for existing drugs beyond their original approved indications.^18^, ^19^ It offers a lower‑risk, faster, and more cost‑effective alternative to de novo development.^20^ In the present study, we screened a commercially acquired library of 950 human endogenous metabolite compounds and identified 4‐Hydroxytamoxifen (4‐OHT) as a candidate drug for LUAD treatment. 4‐OHT, a bioactive metabolite of tamoxifen, is well established as an Estrogen Receptor (ER) modulator in breast cancer.^21^ However, accumulating evidence has suggested that 4‐OHT may have potential anti‐cancer effects beyond breast cancer.^22^ Some studies have reported its cytotoxicity in various cancer cell lines, including those of ovarian, endometrial, and even certain ER‐negative tumours.^23^, ^24^, ^25^, ^26^, ^27^ In the context of lung cancer, although some studies have reported the tumour‐suppressive effects of 4‐OHT, its precise mechanism remains elusive.^28^

Here, we report three major findings: (1) 4‑OHT directly binds to and inhibits AKR1B10, inducing noncanonical ferroptosis in LUAD independently of ER; (2) AKR1B10 sustains LUAD survival through a dual mechanism‐stabilising ACC1 to support de novo lipogenesis and detoxifying cytotoxic reactive carbonyl species (RCSs) such as 4‑HNE‐and its inhibition triggers a metabolic crisis of concurrent lipid deficiency and RCSs accumulation; (3) Nanatinostat, an HDAC inhibitor, similarly targets AKR1B10 and elicits the same cell death modality, providing a rationale for combination with carboplatin or paclitaxel‐based chemotherapy. This study may provide a basis for further exploration of 4‑OHT and AKR1B10 as a potential therapeutic strategy in LUAD.

## 2 MATERIALS AND METHODS

### 2.1 Cell culture

HEK293T, MCF‐7, and HT‐29 cells were cultured in DMEM (Procell, PM150210), while mouse LLC cells and human LUAD cell lines A549, H1299, HCC827, and PC9 were maintained in RPMI‐1640 (Procell, PM150110); both media were supplemented with 10% FBS (Procell, 164210). HEK293T, HLE and LM3 cells were kindly provided by Prof. Jianbin Wang from Nanchang University, Wild‐type HCC827, osimertinib‐resistant HCC827‐OR cells and HT‐29 cells were kindly provided by Prof. Ying Ying from Nanchang University, and wild‐type PC9 and osimertinib‐resistant PC9‐OR cells were kindly provided by Prof. Xiaolei Li from Nanchang University. These osimertinib‐resistant cell lines were cultured in RPMI‐1640 (Procell, PM150110) containing 10% FBS (Procell, 164210). All cell lines were incubated at 37°C in a humidified 5% CO_2_ atmosphere. All cell lines were authenticated by STR profiling and tested negative for mycoplasma contamination. The detailed information is provided in Table S8.

### 2.2 CCK‐8 assay

Cells were seeded at appropriate densities in 96‑well plates, incubated overnight, and treated with indicated compounds. CCK‑8 reagent (Abmole, M4839) was added following treatment, and absorbance was read at 450 nm. Each experiment was performed in triplicate.

### 2.3 Cell growth assay

For cell growth analysis, cells (5000/well) were plated in 24‑well plates, with medium refreshed every 48 h. Cell numbers were quantified on days 0, 2, 4, and 6 by crystal violet staining (absorbance at 595 nm). For colony formation, cells (800/well) were cultured in 6‑well plates with 10% FBS for 10 days, followed by fixation and staining.

### 2.4 Cell death assay

Cell death was assessed by PI (MCE, HY‐D0815) staining followed by flow cytometry. Cells were plated in 6‐well plates (5×10^5^/well) and treated accordingly. Following treatment, cells were harvested, washed with cold PBS, and incubated with PI for 30 min at 37°C in the dark. PI‑positive cells were immediately analysed by flow cytometry (Beckman Coulter, B75442).^29^

### 2.5 Cell cycle profile analysis

For cell cycle profiling, harvested cells were fixed with 70% ethanol (Xilong scientific, 1280340101602), washed, and stained with PI/RNase A staining solution, and analysed by flow cytometry (Beckman Coulter).

### 2.6 Apoptosis assay

After the respective treatment, both the culture supernatant and the trypsinised adherent cells were collected. Cells (5 × 10^5^/well in 6‑well plates) were harvested along with supernatants, washed with PBS, and resuspended in binding buffer. Cells were stained with Annexin V‑FITC and PI (Elabscience, E‑CK‑A211) for 15 min at room temperature in the dark, then immediately analysed by flow cytometry (Beckman Coulter).

### 2.7 Western blotting

Cells were lysed in RIPA buffer (Beyotime, P0013) with protease inhibitors (MCE, HY‑B0496) on ice for 30 min. Lysates were clarified by centrifugation (12 000 × *g*, 20 min, 4°C). Supernatants were mixed with 2× SDS loading buffer and denatured at 100°C for 10 min. Proteins were resolved by 10% SDS‑PAGE, transferred to PVDF membranes (Millipore, IPVH00010), and blocked with 5% non‑fat milk for 1 h. Membranes were probed with primary antibodies overnight at 4°C (details in Table S8), followed by HRP‑conjugated secondary antibodies for 1 h at room temperature. Signals were detected using an ECL kit (Yeasen, 36222ES60) and imaged with a TANON 5500 Gel Imaging System.^30^ Primary antibody information is listed in Table S8. Band intensities were quantified using Image J with normalisation to Vinculin loading controls; representative images were selected from at least three independent experiments with consistent results.

### 2.8 BODIPY staining to assess lipid peroxidation

Lipid peroxidation was assessed using BODIPY 581/591 C11 staining. Treated cells were harvested, washed, and incubated with the probe (Beyotime, S0043S) at 37°C for 30 min, followed by flow cytometric analysis.

### 2.9 CRISPR‒Cas9‐mediated gene knockout

For gene knockout, sgRNAs targeting ERα, ERβ, or AKR1B10 were designed using the online CRISPR tool (https://chopchop.cbu.uib.no/) and cloned into lentiCRISPR‑v2‑Flag. Lentiviral particles were generated by transfecting HEK‑293T cells with the sgRNA construct along with packaging plasmids (pSPAX2 and pMD2.G) using jetPRIMER^®^ (Polyplus). Viral supernatants were harvested, filtered (0.45 µm), and used to transduce target cells. Infected cells were selected with puromycin (10 µg/mL; MCE, HY‑K1057), and knockout efficiency was confirmed by western blotting.

### 2.10 Gene overexpression

Cells at 70%–80% confluency were transfected with designated plasmids (pCMV‐AKR1B10 (human)‐3×FLAG‐Neo, pCMV‐3×FLAG‐Neo) (Miaoling, P49467) using jetPRIMER^®^ transfection reagent (Polyplus Transfection, 101000046). Transfection efficiency was evaluated by western blotting after 48 h.

### 2.11 Untargeted metabolomics

Untargeted metabolomics was performed and analysed by Shanghai Bioprofile Technology Company. Untargeted metabolomics was performed using a UHPLC‐Q‐Exactive Plus‐MS (Thermo Fisher) with an HSS T3 column, operating in ESI ± mode (*m*/*z* 70–1050). Metabolites were identified by mass accuracy (< 10 ppm) against HMDB/MassBank databases. Significantly differential metabolites were filtered by VIP ≥ 1.0 (OPLS‐DA), fold change ≥ 1.5 or ≤ 0.67, and *p* < .05 (Student's *t*‐test) with FDR correction (Benjamini–Hochberg). Lipids were annotated based on HMDB superclass classification. Detailed information is provided in Table S2.

### 2.12 Detection of intracellular ROS

Intracellular ROS levels were assessed using DCFH‑DA (MCE, HY‑D0940). Cells seeded in 24‑well plates (1 × 10^4^/well) and treated as indicated were incubated with 20 µM DCFH‑DA in serum‑free medium for 30 min at 37°C in the dark. After washing, fluorescence images were captured (FITC filter) and quantified using ImageJ.

### 2.13 Lactate dehydrogenase (LDH) release assay

The cell cytotoxicity was evaluated by measuring LDH release. Cells (1 × 10^4^/well) in 96‑well plates were treated as indicated, and LDH activity in culture supernatants was measured using a commercial kit (MCE, HY‑K1090). Fluorescence was recorded on a Varioskan LUX microplate reader, and LDH release was expressed as a percentage of the maximum release from lysed control cells.

### 2.14 GSH/GSSG (glutathione/glutathione disulphide) measurement

Cellular GSH and GSSG levels were assessed using a commercial colorimetric assay kit (Beyotime, S0053). Cells were harvested, washed with PBS, and lysed. After centrifugation, the supernatant was collected for total glutathione and GSSG analysis. To measure GSSG specifically, GSH was masked by derivatisation with 2‐vinylpyridine prior to the assay. The GSH/GSSG ratio was calculated by subtracting GSSG from total glutathione.

### 2.15 Measurement of free fatty acid

The cells were cultured at 37°C for 1 h and then centrifuged at 3000 × *g* for 10 min, following the manufacturer's instructions (Beyotime, S0215S), and the infranatant was collected and used for free fatty acid measurement.

### 2.16 Measurement of malondialdehyde (MDA)

MDA levels were assessed by the Lipid Peroxidation MDA Assay Kit (Beyotime, catalogue number S0131S) following instructions. Absorbance was measured at 532 nm to determine sample concentrations, and results are presented relative to the concentration in control cells.

### 2.17 Iron content analysis

Intracellular iron levels were monitored using Phen Green SK (PGSK, MCE, HY‐126823) diacetate, a dye whose fluorescence is quenched upon interaction with Fe^2^^+^. Cells were incubated with 2 µM PGSK in PBS for 30 min at 37°C. Fluorescence was monitored at 488/525 nm by flow cytometry (Beckman Coulter).

### 2.18 AKR1B10 enzymatic activity assay

AKR1B10 enzymatic activity was measured in HEK293T cells overexpressing pCMV‑AKR1B10‑3×FLAG. Cells were lysed in RIPA buffer for 30 min, and clarified lysates (12 000 × *g*, 10 min, 4°C) were quantified by BCA assay (Beyotime, P0010S). For each reaction, 50 µg of protein was incubated with 125 mM sodium phosphate (pH 7.0), 0.2 mM NADPH (Beyotime, ST360), 50 mM KCl, and 20 mM DL‑glyceraldehyde at 35°C for 10 min. NADPH oxidation was monitored at 340 nm using a microplate reader. For inhibition assays, 4‑OHT, NANA, or epalrestat (0.1–5 µM; MCE, HY‑66009) was pre‑incubated with the enzyme for 5 min before substrate addition.

### 2.19 Protein stability and proteasomal degradation assays

For protein stability, cells were treated with cycloheximide (CHX) at 50 µg/mL and collected at sequential time points. For proteasome inhibition, cells were treated with MG132 (10 µM). ACC1 and GPX4 protein levels were determined by Western blotting.

### 2.20 Drug synergy analysis

For synergy assessment, cells were treated with 4‑OHT combined with carboplatin or paclitaxel for 72 h, and viability was measured by CCK‑8 assay. Synergy was analysed using the ZIP model and Chou–Talalay CI method^31^ (CI < 0.7: strong synergy; 0.7–0.9: moderate; > 0.9: additive), with results visualised via SynergyFinder.

### 2.21 Survival analysis using public datasets

The prognostic relationship between AKR1B10 mRNA expression and overall survival was assessed via the KM‑plotter online platform (https://kmplot.com/analysis/) using two independent cohorts, including a TCGA lung cancer cohort and a cohort enriched for drug‐resistant lung cancer specimens. Patient grouping was defined by the automatically optimised cutoff value of AKR1B10 expression. To explore the association of AKR1B10 with chemotherapy resistance, correlation analysis between AKR1B10 and chemotherapy resistance‐related genes (TUBB3, TYMS, RRM1) was conducted using the GEPIA2 online platform (http://gepia2.cancer‐pku.cn/) with Pearson correlation coefficient calculation.

### 2.22 Limited proteolysis‐mass spectrometry (LiP‐MS)

APTBIO Co., Ltd. (Shanghai, China) conducted the LiP‐MS tests. Detailed QC parameters for the LiP‐MS analysis are as follows: (1) The DIA‐based proteomics analysis was performed on an Orbitrap Astral mass spectrometer with a data‐independent acquisition (DIA) strategy; (2) A total of 3306 protein groups and 35 906 unique peptides were identified across all samples (control, low‐dose 4‐OHT (10 µM), and high‐dose 4‐OHT (20 µM), each in triplicate); (3) Protein false discovery rate (FDR) was controlled at *Q*‐value < 0.01 (equivalent to FDR < 1%); (4) the coefficient of variation between technical replicates was 8.7%, demonstrating high reproducibility; (5) iRT peptide retention times were stable across all runs, confirming the robustness of the LC‐MS/MS system. The detailed information is summarised in Tables S3 and S4.

### 2.23 Surface plasmon resonance (SPR)

The SPR experiment was performed and analysed by MCE.

### 2.24 Animal assay

All animal experiments were approved by the Institutional Animal Care and Use Committee of The First Affiliated Hospital of Nanchang University. Male C57BL/6J mice (4–5 weeks old) and BALB/c‑Nude mice (4 weeks old) were obtained from Jiangsu Jicui Pharmachem Biotechnology Co., Ltd. and maintained under specific pathogen‑free conditions.

For tumour volume monitoring, calliper measurements were taken regularly, and tumour volumes were calculated as V (mm^3^) = (π/6) × length × width^2^. Mice were euthanised when tumours in the vehicle group reached 1500 mm^3^, and tumours were excised, photographed, and weighed. Investigators performing tumour measurements and data analysis were blinded to group assignment. Animals without palpable tumours by day 14 post‑inoculation were excluded prior to randomisation; all remaining animals were included in the final analysis.

To establish the LLC tumour model, Lewis lung carcinoma (LLC) cells (4 × 10^5^ in 100 µL) were inoculated subcutaneously into the flank of each mouse. 14 days later, the mice were randomly allocated into groups: (1) Vehicle control; 4‐OHT group (20 mg/kg, once a day); 4‐OHT group (60 mg/kg, once a day). (2) Vehicle control; 4‐OHT group (60 mg/kg, once a day); carboplatin (Carbo) (20 mg/kg, twice a week) (MCE, catalogue number HY‐17393); the combination of 4‐OHT (60 mg/kg) and Carbo (20 mg/kg).^32^

To establish the H1299 subcutaneous xenograft models in BALB/c‐Nude mice, four stably engineered H1299 cell lines (sg#NC, sg#AKR1B10, oe#NC, oe#AKR1B10) were prepared for subcutaneous implantation (5 × 10^6^ cells in 100 µL). Starting two weeks after cell injection, mice were intraperitoneally injected with 60 mg/kg 4‐OHT or matching DMSO vehicle once daily for one week. Four monotherapy groups were generated for subsequent phenotype detection: sg#NC + 4‐OHT, sg#AKR1B10 + 4‐OHT, oe#NC + 4‐OHT, and oe#AKR1B10 + 4‐OHT.

### 2.25 Immunohistochemistry (IHC)

A LUAD tissue microarray was obtained from the National Engineering Center for BioChips (Shanghai Biochip) and Servicebio, with specimen details in Table S5. Sections were deparaffinised, rehydrated, and subjected to antigen retrieval, followed by quenching of endogenous peroxidase with 3% H_2_O_2_ and blocking. The sections were then incubated with anti‑AKR1B10 antibody (Abclonal, A7823) overnight at 4°C, washed, and incubated with HRP‑conjugated secondary antibody for 1 h. Immunoreactivity was developed using DAB, and counterstained with haematoxylin. Images were captured on an Olympus IX71 microscope and quantified with ImageJ.

IHC scoring was performed by two pathologists blinded to clinical data. Staining intensity was graded as 0 (negative) to 3 (strong), and the percentage of positive cells as 0 (0%) to 4 (76%–100%). The final score (intensity × proportion, range 0–12) classified specimens as high (≥ 4) or low (< 4) AKR1B10 expression.

### 2.26 Transmission electron microscopy (TEM) assay

For TEM analysis, cells were seeded at 2 × 10^6^ per dish, collected, and fixed with 2.5% glutaraldehyde in 0.1 M phosphate buffer. Fixed samples were then submitted to Servicebio (Wuhan, China) for post‑fixation, dehydration, resin embedding, ultrathin sectioning, counterstaining, and imaging. Representative TEM images were acquired.

### 2.27 Cellular 4‐HNE measurements

Cells were treated with 20 µM 4‐OHT for 48 h in 10 cm dishes, then scraped into 500 µL ddH_2_O. Cell suspension was vigorously mixed with 45% perchloric acid. After centrifugation (14 000 rpm, 4°C, 10 min), the supernatant was collected for high‐performance liquid chromatography (HPLC) analysis.

### 2.28 Streptavidin magnetic beads pull‐down assay

Pull‑down assays were conducted using streptavidin‑coated magnetic beads (MCE, HY‑K0208). Cells were lysed in buffer with protease inhibitors, and clarified supernatants were incubated with biotinylated 4‑OHT and beads for 6 h at 4°C. For competition experiments, a 10‑fold excess of unlabelled 4‑OHT (100 µM) was added. After magnetic capture and washing, bound proteins were eluted by boiling in SDS loading buffer and analysed by western blotting.

### 2.29 Molecular docking

Protein structures were obtained from the Research Collaboratory for Structural Bioinformatics (RCSB) database, while compound structures were sourced from PubChem. Molecular docking procedures and interaction analyses were performed with AutoDock 4.2 (The Scripps Research Institute, La Jolla, CA, USA).

### 2.30 RNA extraction and qRT‐PCR

Total RNA was isolated with Liji Biotechnology RNA extraction reagent. Purified RNA was reverse‐transcribed into cDNA via Vazyme kits for mRNA detection, with all primer sequences provided in Table S8. qPCR was performed using Vazyme ChamQ SYBR Green master mix on an Applied Biosystems QuantStudio 5 platform. GAPDH was utilised as the reference gene for mRNA normalisation. Relative mRNA expression was calculated using the 2^−^ΔΔCt method.

### 2.31 Statistical analysis

All data are presented as mean ± SD from a minimum of three independent replicates. Group comparisons were performed using either two‑tailed unpaired Student's *t*‑test (for two groups) or one‑way ANOVA with Tukey's post hoc test (for multiple groups). IC_50_ values were derived by nonlinear regression fitting a four‑parameter logistic model.

Significance was set at *p* < .05, with ns (not significant) for *p* > .05, and *p < .05, **p < .01, ***p < .001. FlowJo was used for flow cytometry data processing, and GraphPad Prism 10.0 was used for graphing.

## 3 RESULTS

### 3.1 4‐OHT inhibits the viability of LUAD cells in an ER‐independent manner

**FIGURE 1:**
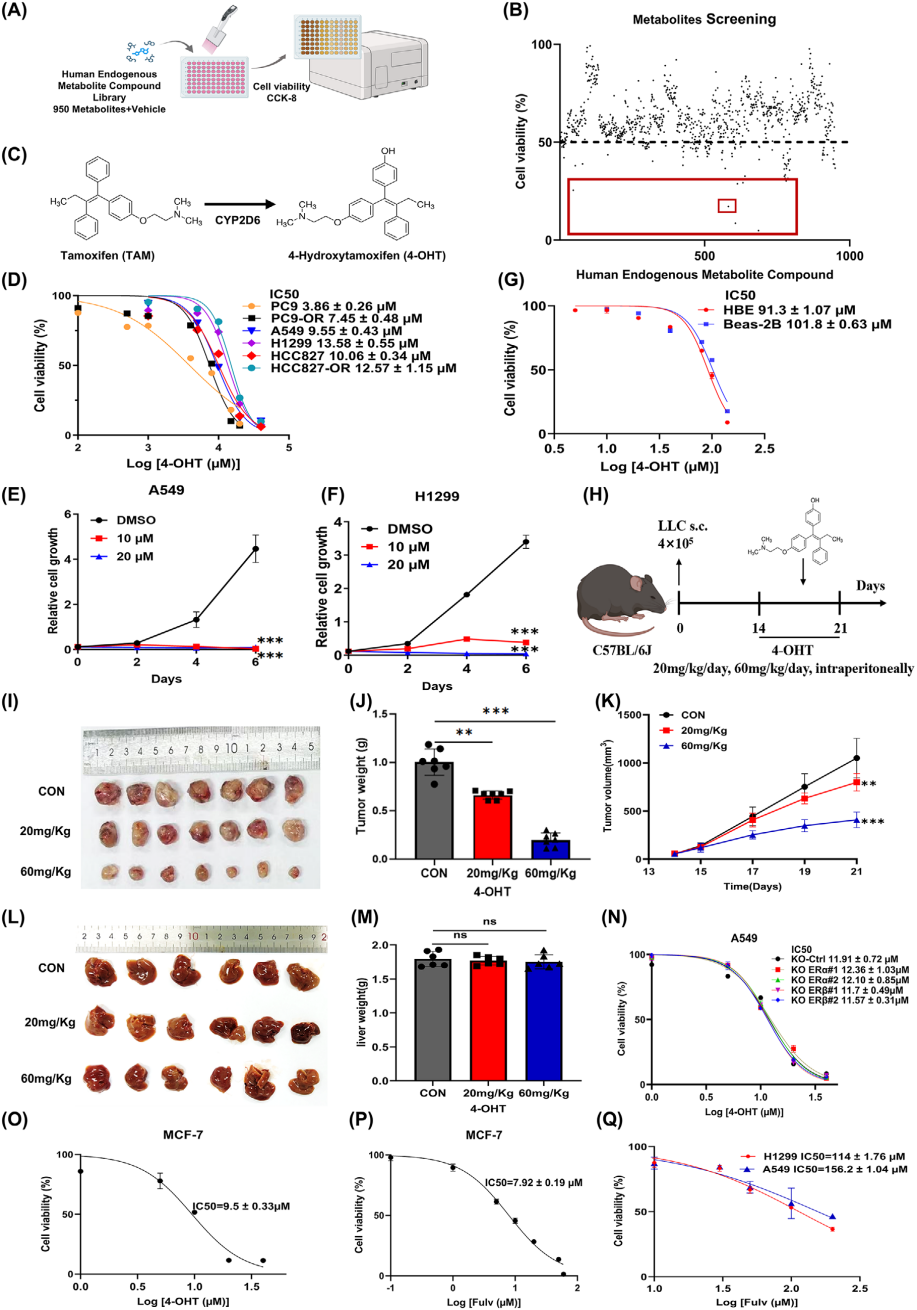
4‐OHT inhibits the viability of LUAD cells in an ER‐independent manner. (A) Schematic workflow of the drug repurposing screen. PC9 cells were treated with 950 human endogenous metabolite compounds (10 µM, 24 h), and cell viability was assessed by CCK‐8 assay. (B) Cell viability screening results in PC9 cells. The red box highlights 4‐hydroxytamoxifen (4‐OHT). (C) Chemical structure of 4‐OHT, the active hepatic metabolite of tamoxifen. (D) Dose–response curves of LUAD cell lines treated with 4‐OHT (0‐40 µM, 72 h). Cell viability assessed by CCK‐8 assays. The 50% inhibition concentration (IC_50_) values were calculated by nonlinear regression (mean ± SD, *n* = 3, two‐way ANOVA). (E and F) A549 (E) and H1299 (F) cells were treated with 4‐OHT, and cell proliferation was assessed by crystal violet staining at the indicated time (mean ± SD, *n* = 3, *** *p* < .001, two‐way ANOVA). (G) Cytotoxicity of 4‐OHT in normal lung epithelial cells (HBE and Beas‐2B) at 72 h. The IC_50_ values were calculated by nonlinear regression (mean ± SD, *n* = 3, two‐way ANOVA). (H) Schematic of the syngeneic allograft model. C57BL/6J mice received subcutaneous injection of LLC cells (4 × 10^5^ cells). After 14 days, mice were treated with 4‐OHT (20 or 60 mg/kg, i.p., daily, *n* = 7 per group) for 7 days. (I) Representative tumour images at endpoint. (J) Tumour weights at endpoint (mean ± SD, *n* = 7, **p < .01, ***p < .001, one‐way ANOVA). (K) Tumour volumes measured over time (mean ± SD, *n* = 7, **p < .01, ***p < .001, two‐way ANOVA). (L and M) Representative liver images (L) and liver weights (M) at endpoint (mean ± SD, *n* = 6, ns, not significant, *p* > .05, one‐way ANOVA). (N) ER knockout does not affect 4‐OHT sensitivity. A549 cells with CRISPR‐Cas9‐mediated knockout of ERα/ERβ were treated with 4‐OHT for 72 h (mean ± SD, *n* = 3, two‐way ANOVA). (O and P) MCF‐7 cells treated with 4‐OHT (O) or fulvestrant (P) for 72 h. IC_50_ values were calculated by nonlinear regression (mean ± SD, *n* = 3, two‐way ANOVA). (Q) A549 and H1299 cells treated with fulvestrant for 72 h. IC_50_ values were calculated by nonlinear regression (mean ± SD, *n* = 3, two‐way ANOVA).

Through a drug repurposing screen of 950 purchased human endogenous metabolic compounds (Table S1), we initially identified agents exhibiting 50% tumour growth inhibition using a CCK‐8 assay in PC9 cells (Figure 1A and B). Following a literature review of several promising candidates, we determined that the mechanism underlying the role of 4‐Hydroxytamoxifen (4‐OHT), the hepatic metabolite of tamoxifen, remains unclear in LUAD (Figure 1C).^22^ The wild‐type LUAD cells (PC9, A549, H1299 and HCC827) and osimertinib‐resistant LUAD cells (PC9‐OR, HCC827‐OR) were used to investigate the inhibitory effect of 4‐OHT (Figure S1A–D). The 50% inhibition concentration (IC_50_) of 4‐OHT ranged from 3.86 ± 0.26 µM to 13.58 ± 0.55 µM in the cells we tested (Figure 1D). Crystal violet staining and clonogenic assays demonstrated that 4‐OHT significantly suppressed the proliferation of LUAD cells in vitro (Figures 1E and F and S1E–I). However, the IC_50_ of 4‐OHT in human bronchial epithelial cells were 101.8 ± 0.63 µM (Beas‐2B) and 91.3 ± 1.07 µM (HBE), indicating a relatively low toxicity to normal cells (Figure 1G). Additionally, the antitumour activity of 4‐OHT was assessed in vivo using a Lewis lung carcinoma cell (LLC) model in C57BL/6J mice. Similarly, 4‑OHT also markedly suppressed LLC cell proliferation in vitro (Figures 1H and S1J–L). Daily administration of 4‐OHT (20 mg/kg or 60 mg/kg) significantly suppressed tumour growth compared with the vehicle control group, especially for the 60 mg/kg group (Figure 1I–K). Body and liver weights remained unchanged at both concentrations, indicating tolerable cytotoxicity (Figures 1L and M and S1M). Collectively, these findings demonstrate that 4‐OHT effectively inhibits LUAD progression both in vitro and in vivo.

Given that 4‐OHT primarily targets the estrogen receptor (ER) for breast cancer treatment, we wondered if 4‐OHT also targets ER in LUAD.^33^ Firstly, we assessed the expression of estrogen receptor alpha (ERα) and beta (ERβ) in LUAD cell lines. The results revealed the heterogeneous ER expression profiles across different LUAD cell lines, especially for ERα (Figure S1N). Subsequently, we performed CRISPR‐Cas9‐mediated ER knockout, and there were no significant changes in the sensitivity of LUAD cells to 4‐OHT (Figures 1N and S1O and P). Interestingly, pharmacological modulation of ER signalling, or using the ER antagonist fulvestrant, significantly inhibited the viability of ER+ breast cancer cell MCF‐7 (Figure 1O and P). A549 and H1299 cells showed a markedly reduced sensitivity to fulvestrant, requiring roughly 20‐fold higher concentrations to achieve similar inhibition (Figure 1Q). These findings imply that the anti‐tumour activity of 4‐OHT in LUAD may not be mediated through the classical ER pathway, suggesting the potential existence of novel cellular targets.

### 3.2 Compared with ER‐positive breast cancer cells, 4‐OHT preferentially induces cell death in LUAD cells

**FIGURE 2:**
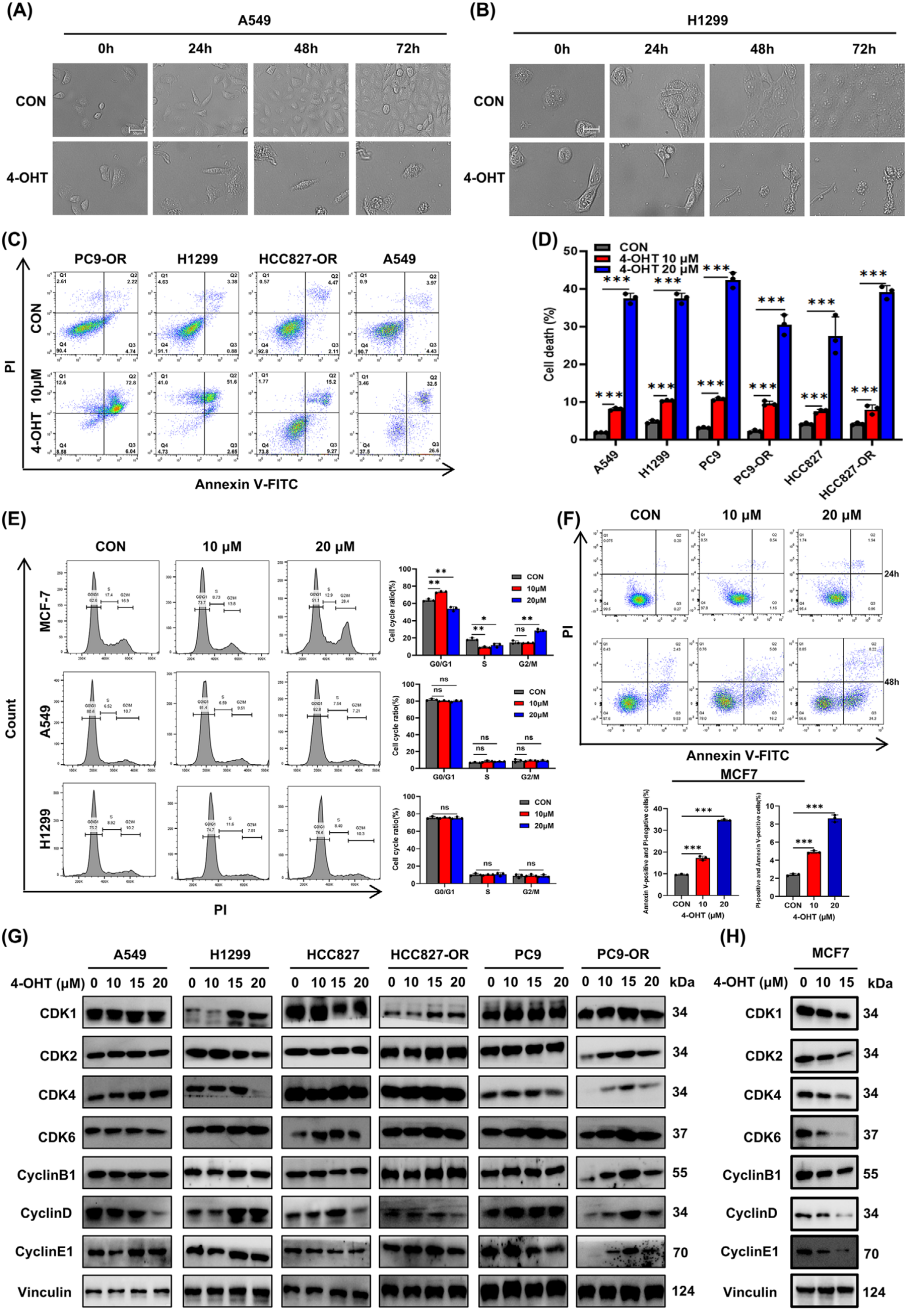
Compared with ER‐positive breast cancer cells, 4‐OHT preferentially induces cell death in LUAD cells. (A and B) Time‐lapse imaging of A549 (A) and H1299 (B) cells treated with 4‐OHT (20 µM) or vehicle (DMSO) over 72 h. Scale bars: 50 µm. Representative images from three independent experiments are shown. (C) Flow cytometry analysis of apoptosis in A549, H1299, HCC827‐OR, and PC9‐OR cells treated with 4‐OHT (20 µM) or control for 48 h. Cells were stained with Annexin V‐FITC and propidium iodide (PI). Representative plots from three independent experiments are shown. (D) Quantification of cell death in LUAD cell lines treated with 4‐OHT (10 or 20 µM) or control for 48 h. Cell death was assessed by PI staining and flow cytometry (mean ± SD, *n* = 3, ***p < .001, two‐tailed unpaired Student's *t*‐test). (E) Cell cycle distribution in MCF‐7, A549, and H1299 cell lines was analysed by flow cytometry 24 h after treatment with 4‐OHT (mean ± SD, *n* = 3, ns, not significant, *p* > .05; *p < .05; **p < .01; ***p < .001, two‐way ANOVA with Tukey's post hoc test). (F) Time‐course analysis of MCF‐7 cells treated with 4‐OHT (20 µM) or control for 24 or 48 h. Left panel: percentage of Annexin V^+^/PI^−^ cells (early apoptosis); right panel: percentage of Annexin V^+^/PI^+^ cells (late apoptosis) (mean ± SD, *n* = 3, ***p < .001, two‐tailed unpaired Student's *t*‐test). (G) Western blot analysis of cell cycle regulatory proteins (CDK1, CDK2, CDK4, CDK6, cyclin B1, cyclin D, cyclin E1) in LUAD cell lines (A549, H1299, PC9, PC9‐OR, HCC827, and HCC827‐OR) treated with 4‐OHT (10 µM, 15 µM, or 20 µM) for 24 h. Vinculin served as a loading control. Representative blots from three independent experiments are shown. (H) Western blot analysis of cell cycle regulatory proteins in MCF‐7 cells treated with 4‐OHT (10 µM or 15 µM) for 24 h. Vinculin served as a loading control. Representative blots from three independent experiments are shown.

To further elucidate the mechanism by which 4‐OHT inhibits LUAD, continuous cell monitoring revealed that prolonged (72 h) exposure induced significant cellular lysis (Figure 2A and B). Subsequently, we detected cell death using Annexin‐V/PI double staining method. Interestingly, a large proportion of cells exhibited Annexin‐V/PI double staining and the proportion of these cells exceeded that of Annexin‐V single stained cells, indicating that 4‑OHT triggered substantial cell death in LUAD cells (Figure 2C). We also examined cell death using PI single staining followed by flow cytometry. Figure 2D showed that 4‐OHT treatment induced significant cell death in all tested LUAD cell lines. We then performed cell cycle analysis in MCF‐7 and LUAD cells following short‐term 4‐OHT treatment (24 h). Quantitative analysis revealed that 4‐OHT treatment induced cell cycle arrest in ER‐positive MCF‐7 cells, as reported previously.^33^, ^34^ In contrast, cell cycle profiles of the LUAD cells (A549 and H1299) remained largely unaffected, with no statistically significant changes in the distribution of cells across different phases (Figure 2E). We then detected 4‐OHT‐induced apoptosis in MCF‐7 cells. The percentage of Annexin‑V^+^/PI^−^ cells in MCF‑7 cultures rose sharply from 24 h to 48 h of 4‑OHT treatment, while PI single‑positive cells remained scarce at both time points (Figure 2F). Annexin‑V^+^/PI^+^ cells increased at 48 h but remained far fewer than Annexin‑V^+^/PI^−^ cells (Figure 2F). This observation is consistent with the cell cycle arrest induced by short‐term (24 h) 4‐OHT treatment in MCF‐7 cells, whereas prolonged treatment led to apoptosis (Figure 2E and F). Furthermore, we examined cell cycle‐related protein expression in LUAD cells. Figures 2G and S2A–G show that 4‐OHT did not induce a consistent trend of changes in cyclins and CDKs. By contrast, cyclins and CDKs were markedly decreased in MCF‐7 cells (Figures 2H and S2H–N). Collectively, these findings demonstrate that 4‐OHT preferentially induces cell death in LUAD cells, rather than inhibiting proliferation through cell cycle arrest. Given that 4‐OHT predominantly induces cell death in LUAD cells, we next characterised the molecular nature of this cell death.

### 3.3 4‐OHT induces a noncanonical form of cell death

**FIGURE 3:**
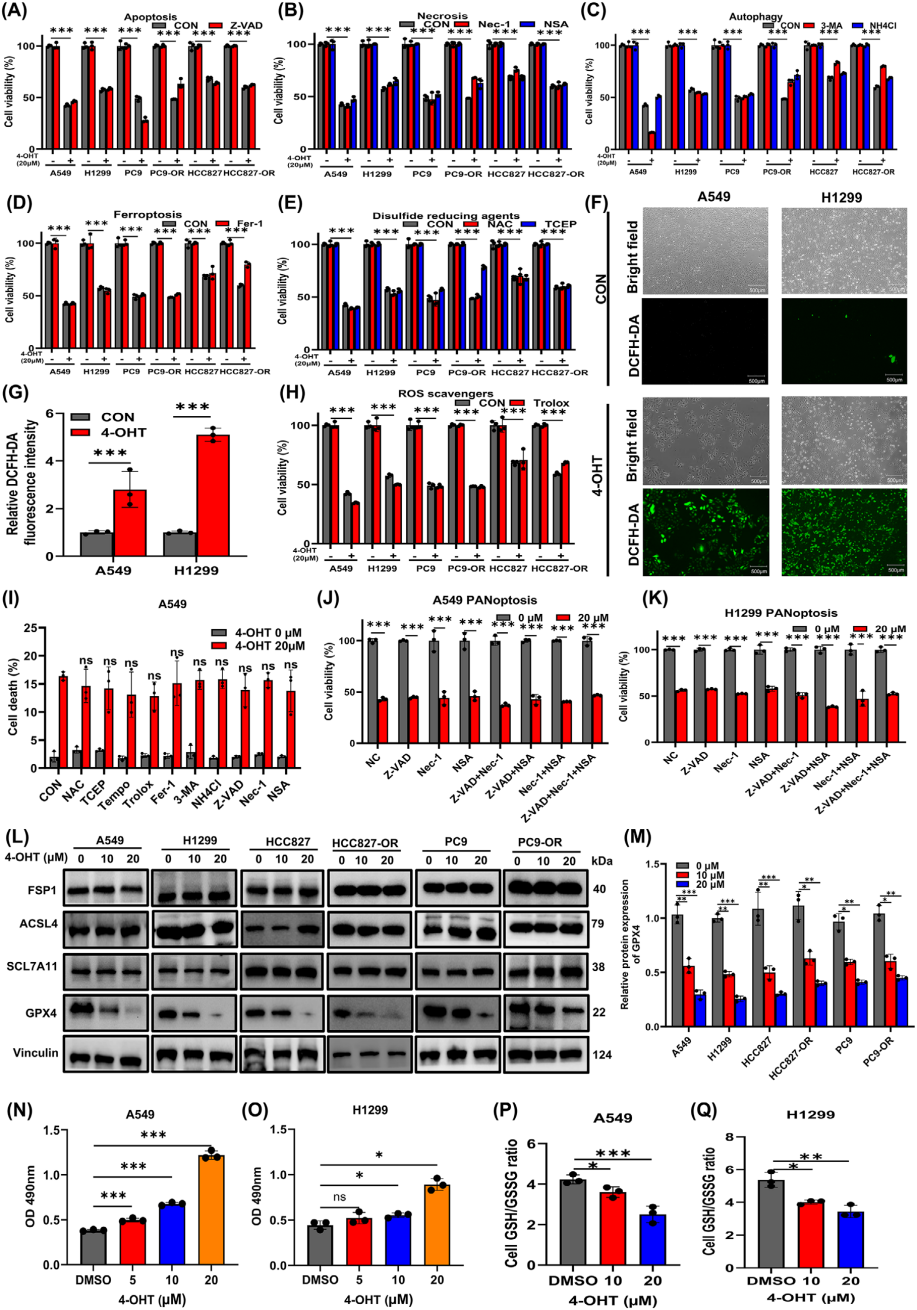
4‐OHT induces a noncanonical form of cell death. (A–E) Cell viability assessed by CCK‑8 assay after 48 h treatment with 4‑OHT in the presence of inhibitors of apoptosis (Z‑VAD‑FMK, A), necroptosis (Nec‑1 and NSA, B), autophagy (3‑MA and NH_4_Cl, C), ferroptosis (Fer‑1, D), or disulphidptosis (NAC and TCEP, E). None of these inhibitors rescued 4‑OHT‑induced cell death (mean ± SD, *n* = 3, ***p < .001, two‑way ANOVA with Tukey's post hoc test). (F) Representative images of intracellular ROS levels in A549 and H1299 cells treated with 4‑OHT (10 µM) for 24 h, stained with DCFH‑DA. Scale bars: 500 µm. Representative images from three independent experiments are shown. (G) DCFH‑DA fluorescence intensity was measured and normalised to control (mean ± SD, *n* = 3, ***p  <  .001, two‑tailed unpaired Student's *t*‑test). (H) Cells were treated with 4‐OHT and the ROS scavenger Trolox. At 48 h after treatment, cell viability was assessed using the CCK‐8 assay (mean ± SD, *n* = 3, ***p <  .001, two‑way ANOVA with Tukey's post hoc test). (I) 4‑OHT‑induced cell death in A549 cells could not be rescued by various cell death inhibitors, as quantified by PI staining and flow cytometry (mean ± SD, *n* = 3, ns, not significant, *p* > .05, one‑way ANOVA with Tukey's post hoc test). (J and K) PANoptosis inhibitor combinations failed to rescue 4‑OHT‑induced cell death. A549(J) and H1299 (K) cells were treated with the inhibitor Z‑VAD‑FMK, Nec‑1, NSA alone or in combination with 4‑OHT for 48 h. Cell viability was assessed by CCK‑8 assay (mean ± SD, *n* = 3, ***p   <    .001, two‑way ANOVA with Tukey's post hoc test). (L) Western blot analysis of ferroptosis‐related proteins (FSP1, ACSL4, SLC7A11, GPX4) in LUAD cells after treatment with 4‑OHT for 48 h. Vinculin served as a loading control. Representative blots from three independent experiments are shown. (M) Quantification of GPX4 protein levels normalised to Vinculin (mean ± SD, *n* = 3, *p < .05; **p < .01; ***p < .001, two‑tailed unpaired Student's *t*‑test). (N and O) Detection of Lactate dehydrogenase (LDH) release in A549 (N) and H1299 (O) cells treated with 4‐OHT at the indicated time (mean ± SD, *n* = 3, ns, not significant, *p* > .05; *p < .05; ***p  <  .001, two‑way ANOVA). (P and Q) GSH/GSSG ratio in A549 (P) and H1299 (Q) cells treated with 4‐OHT at the indicated time (mean ± SD, *n* = 3, *p < .05; **p < .01; ***p < .001, two‑way ANOVA).

Having established that 4‐OHT exerts its inhibitory effect by inducing cell death in LUAD cells, we further explored which form of programmed cell death 4‐OHT induced. Rescue experiments using specific inhibitors targeting the classical forms of programmed cell death (apoptosis, necroptosis, autophagy, ferroptosis, and disulphidptosis) revealed that none of these inhibitors consistently rescued 4‐OHT‐induced cell death in all tested LUAD cells (Figures 3A–E and S3A–F). Similarly, the ROS scavenger Trolox failed to prevent cell death induced by 4‐OHT, despite a concomitant increase in intracellular ROS levels (Figures 3F–H and S3G). Flow cytometry analysis using PI staining further confirmed that none of the inhibitors effectively blocked 4‐OHT‐induced cell death (Figures 3I and S3H). Moreover, we examined whether PANoptosis, which integrates pyroptosis, apoptosis, and necroptosis, was involved; however, none of the inhibitor combinations tested could rescue cell death (Figures 3J and K and S3I–L). We then detected the expression of the hallmark proteins associated with these cell death pathways. These results did not support apoptosis, necroptosis, autophagic, and pyroptosis cell death as the primary mechanisms underlying 4‐OHT‐induced cell death (Figure S4A–D). For ferroptosis, the expression of FSP1, ACSL4, and SLC7A11 remained unchanged, whereas GPX4 was markedly downregulated in LUAD cells (Figures 3L and M and S3M–O). To determine whether 4‑OHT‑induced GPX4 downregulation occurs at the transcriptional or post‑translational level, we first performed qPCR analysis and found that GPX4 mRNA levels remained unchanged upon 4‑OHT treatment (Figure S3P). We then conducted cycloheximide (CHX) chase assays to examine GPX4 protein stability. As shown in Figure S3Q, GPX4 protein declined gradually in CHX‑treated control cells over 12 h. Notably, 4‑OHT treatment markedly accelerated GPX4 degradation. Importantly, co‑treatment with the proteasome inhibitor MG132 abrogated this 4‑OHT‑induced acceleration. These results suggest that 4‑OHT may promote GPX4 degradation, at least in part, through the ubiquitin‑proteasome pathway.

Furthermore, we also observed a significant elevation of lactate dehydrogenase (LDH) release in LUAD cells, indicating that 4‐OHT‐induced cell membrane damage (Figure 3N and O). Notably, 4‐OHT treatment significantly reduced the GSH/GSSG ratio in both A549 and H1299 cells in a dose‐dependent manner (Figure 3P and Q). These results demonstrate that 4‐OHT‐induced cell death was characterised by GPX4 downregulation, increased ROS levels, and a reduced GSH/GSSG ratio. However, other canonical ferroptosis‐associated proteins showed no significant changes, and the ferroptosis inhibitor Fer‐1 failed to rescue cell death. Collectively, these findings suggest that 4‐OHT induces a noncanonical cell death.

### 3.4 4‐OHT induces lipid deficiency and lipid peroxidation

**FIGURE 4:**
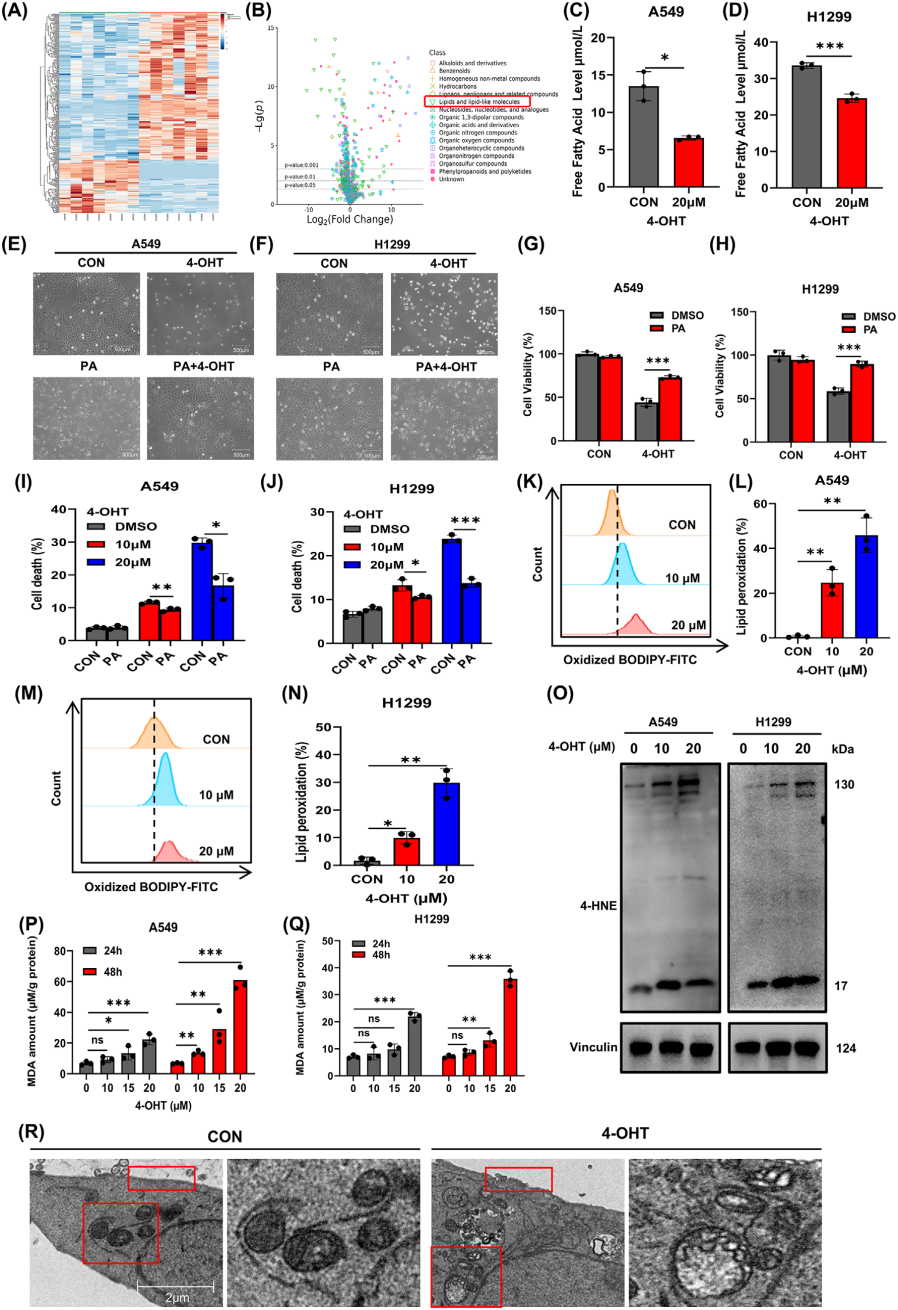
4‐OHT induces lipid deficiency and lipid peroxidation. (A) Heatmap of untargeted metabolomics data showing differential metabolites in A549 cells treated with 4‐OHT (20 µM, 48 h) compared with untreated controls (*n* = 6). (B) Volcano plot of untargeted metabolomics data. The red box highlights lipids and lipid‐like molecules. (C and D) Free fatty acid (FFA) content in A549 (C) and H1299 (D) cells treated with 4‐OHT for 48 h. Absorbance was measured at 570 nm (mean ± SD, *n* = 3, *p  <  .05; ***p  <  .001, one‑way ANOVA with Dunnett's post hoc test). (E and F) Representative images of A549 (E) and H1299 (F) cells treated with 4‑OHT (20 µM) with or without palmitic acid (PA, 25 µM) for 48 h. Scale bars: 500 µm. (G and H) Cell viability of A549 (G) and H1299 (H) cells treated with 4‑OHT with or without PA (25 µM) for 48 h, assessed by CCK‑8 assay (mean ± SD, *n* = 3, ***p < .001, two‑way ANOVA with Tukey's post hoc test). (I and J) Cell death in A549 (I) and H1299 (J) cells treated with 4‑OHT (20 µM) with or without PA (25 µM) for 48 h, quantified by PI staining and flow cytometry (mean ± SD, *n* = 3, *p < .05; **p < .01; ***p < .001, two‑way ANOVA with Tukey's post hoc test). (K–N) Relative lipid peroxidation level in A549 (K and L) and H1299 (M and N) cells when treated with 4‐OHT for 48 h, measured by flow cytometry. Quantification of mean fluorescence intensity is shown (mean ± SD, *n* = 3, *p  <  .05; **p < .01, one‑way ANOVA with Dunnett's post hoc test). (O) Western blotting analysis of 4‐HNE adducts levels in A549 and H1299 cells treated with 4‐OHT for 48 h. Representative blots from three independent experiments are shown. (P and Q) Intracellular malondialdehyde (MDA) levels in A549 (P) and H1299 (Q) cells treated with 4‐OHT for 24 h and 48 h, measured by absorbance at 532 nm (mean ± SD, *n* = 3, ns, not significant, *p* > .05; *p < .05; **p < .01; ***p  <  .001, two‑way ANOVA with Tukey's post hoc test). (R) Representative transmission electron microscopy (TEM) images of A549 cells treated with DMSO or 4‑OHT (20 µM) for 48 h. Scale bars: 2 µm.

To further characterise the features of the cell death induced by 4‐OHT, we performed untargeted metabolomics analysis (Table S2). Significant alterations in metabolite profiles were observed upon 4‐OHT treatment, with a pronounced reduction in lipids and lipid‐like molecules (Figure 4A and B). Consistent with this, the intracellular free fatty acid levels were markedly decreased (Figure 4C and D). These results suggested that fatty acid deficiency may serve as a contributing factor to 4‐OHT‐induced cell death. Importantly, supplementation with palmitic acid (PA), a saturated fatty acid that can be incorporated into cellular lipid pools, effectively rescued 4‐OHT‐induced cell death in both A549 and H1299 cells (Figures 4E–J and S5A–J). Having observed that 4‑OHT elevated intracellular ROS levels, we next examined whether 4‐OHT also induced lipid peroxidation. Figure 4K–N showed that lipid peroxidation was significantly elevated in both A549 and H1299 cells treated with 4‐OHT. 4‐hydroxynonenal (4‐HNE) is a toxic lipid peroxide; we detected the 4‐HNE levels using High Performance Liquid Chromatography (HPLC) and western blotting. The results showed that 4‐OHT treatment significantly elevated both 4‐HNE and 4‐HNE‐modified protein levels (Figures 4O, S5K, and S6A–E). The levels of the toxic lipid peroxide malondialdehyde (MDA) also increased in a dose‐ and time‐dependent manner (Figure 4P and Q). The transmission electron microscopy (TEM) analysis further demonstrated that 4‐OHT treatment induced morphological alterations, characterised by cell membrane rupture and disruption, disappearance of mitochondrial cristae, as well as mitochondrial swelling (Figure 4R). These findings demonstrate that 4‐OHT induces a cell death characterised by lipid deficiency and lipid peroxidation.

### 3.5 4‐OHT induces a noncanonical ferroptosis

**FIGURE 5:**
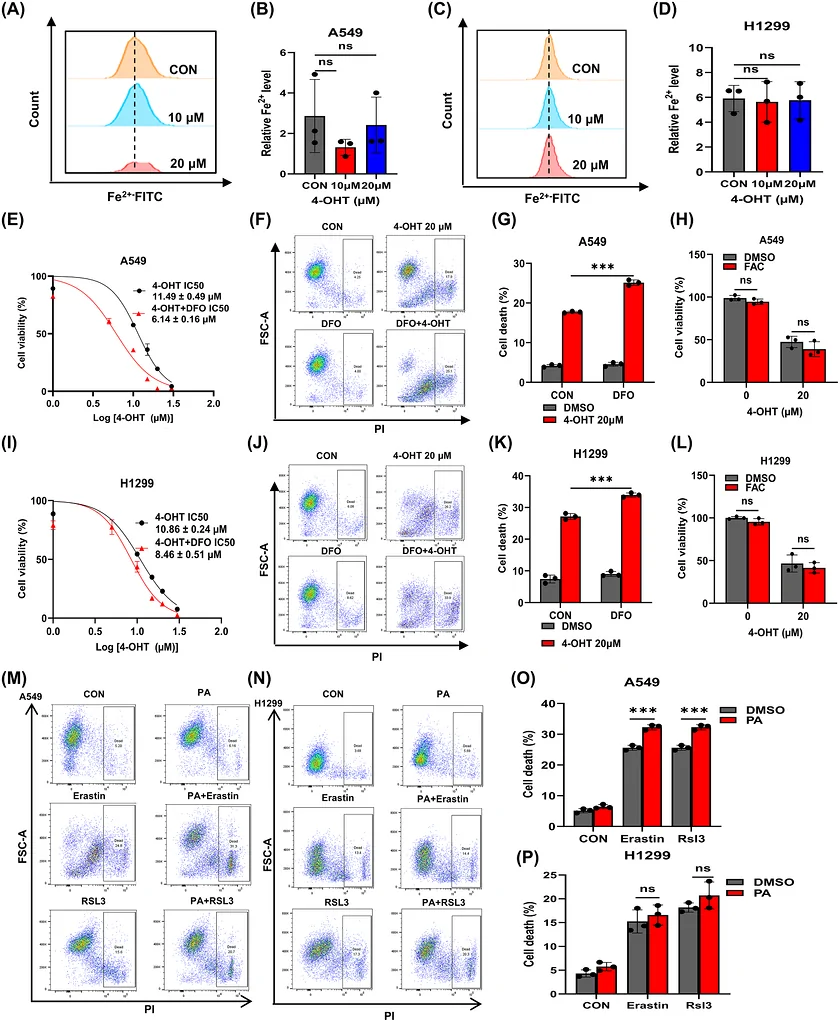
4‐OHT induces a noncanonical ferroptosis. (A–D) Intracellular iron levels in A549 (A, B) and H1299 (C, D) cells treated with 4‑OHT for 48 h, labelled with PGSK and analysed by flow cytometry. Quantification of fluorescence intensity is shown in (B) and (D) (mean ± SD, *n* = 3, ns, not significant, *p* > .05, one‑way ANOVA with Tukey's post hoc test). (E) Cell viability of A549 cells treated with 4‐OHT in the presence of DFO (2 µM) for 72 h. IC_50_ values were calculated by nonlinear regression (mean ± SD, *n* = 3, two‐way ANOVA). (F and G) Cell death in A549 cells treated with 4‑OHT in the presence of DFO (2 µM) for 48 h, quantified by flow cytometry (mean ± SD, *n* = 3, ***p < .001, one‑way ANOVA with Tukey's post hoc test). (H) Cell viability of A549 cells treated with 4‑OHT (20 µM) with or without ferric ammonium citrate (FAC, 100 µM) for 48 h, assessed by CCK‑8 assay (mean ± SD, *n* = 3, ns, not significant, *p* > .05, one‑way ANOVA with Tukey's post hoc test). (I) Cell viability of H1299 cells treated with 4‐OHT in the presence of DFO (2 µM) for 72 h. IC_50_ values were calculated by nonlinear regression (mean ± SD, *n* = 3, two‐way ANOVA). (J and K) Cell death in H1299 cells treated with 4‑OHT in the presence of DFO (2 µM) for 48 h, quantified by flow cytometry (mean ± SD, *n* = 3, ***p < .001, one‑way ANOVA with Tukey's post hoc test). (L) Cell viability of H1299 cells treated with 4‑OHT (20 µM) with or without FAC (100 µM) for 48 h, assessed by CCK‑8 assay (mean ± SD, *n* = 3, ns, not significant, *p* > .05, one‑way ANOVA with Tukey's post hoc test). (M–P) Ferroptosis was induced in A549 (M and O) and H1299 (N and P) cells using Erastin and RSL3. Cell death was quantified by flow cytometry. PA supplementation could not rescue the cell death (mean ± SD, *n* = 3, ns, not significant, *p* > .05; ***p < .001, one‑way ANOVA with Tukey's post hoc test).

To further distinguish 4‐OHT‐induced cell death from canonical ferroptosis, we explored whether this cell death was iron‐dependent.^6^ First, the intracellular iron levels remained unaltered after 4‐OHT treatment in both A549 and H1299 cells (Figure 5A–D). We also examined whether iron participates in 4‐OHT‐induced cell death. Co‐treatment with the iron chelator deferoxamine (DFO) did not attenuate the anti‐tumour efficacy of 4‐OHT in either A549 or H1299 cells (Figure 5E and I). On the contrary, DFO treatment enhanced the inhibitory effects of 4‐OHT on LUAD cells (Figure 5F, G, J, and K). Exogenous supplementation with ferric ammonium citrate (FAC) did not rescue or exacerbate 4‑OHT‐induced cell death in A549 and H1299 cells (Figure 5H and L).

Given that PA treatment inhibited 4‐OHT‐induced cell death, we next examined whether PA treatment also blocked canonical ferroptosis. Ferroptosis was induced using the well‐established ferroptosis inducers RSL3 and Erastin. PA supplementation failed to rescue RSL3‐ or Erastin‐induced ferroptosis, as confirmed by flow cytometry in A549 and H1299 cells (Figure 5M–P). Previous studies have shown that monounsaturated fatty acids (MUFA) suppress ferroptosis,^35^ whereas the concentration of PA required to induce lipotoxicity is typically 500 µM,^36^ substantially higher than the 25 µM used in our rescue experiments. Therefore, the protective effect of low‐dose PA is unlikely to result from non‐specific cytotoxicity or generalised metabolic stress, and instead likely reflects the specific reversal of lipid deficiency. Collectively, these data demonstrate that 4‐OHT induces a noncanonical ferroptosis that is distinct from classical ferroptosis.

### 3.6 4‐OHT induces noncanonical ferroptosis by targeting AKR1B10

**FIGURE 6:**
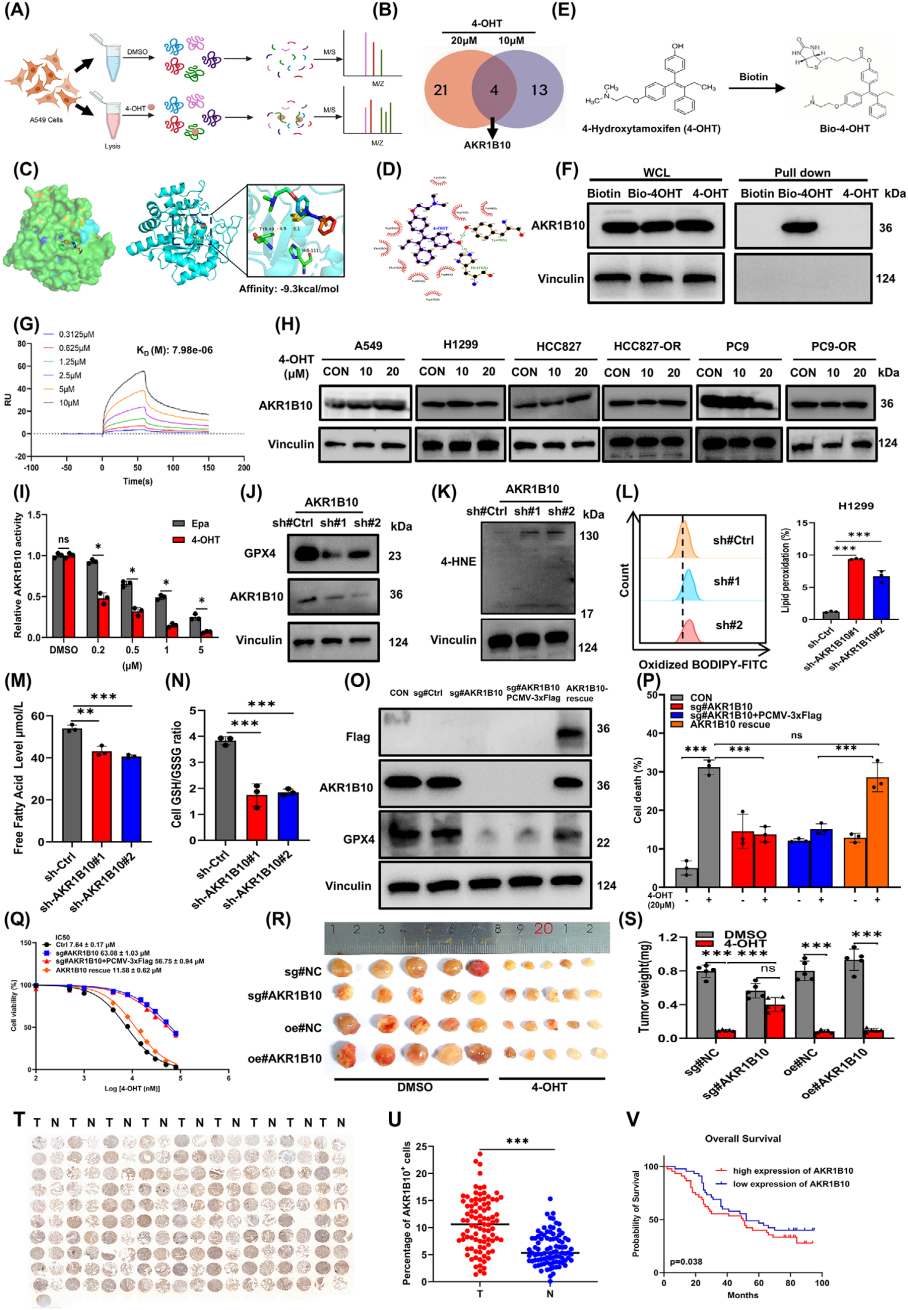
4‐OHT induces noncanonical ferroptosis by targeting AKR1B10. (A) Schematic diagram of the LiP‐MS workflow for identification of the target of 4‐OHT. (B) Venn diagram of proteins with characteristic peptides identified by LiP‐MS in cells treated with different concentrations of 4‑OHT (10 and 20 µM). (C) Molecular docking simulation of the binding between 4‑OHT and AKR1B10. (D) Schematic illustration of the molecular interaction between 4‐OHT and surrounding amino acid residues of AKR1B10, showing the binding mode and key contact sites. (E) Chemical structure of biotinylated 4‐OHT. (F) Pull‑down assay using biotinylated 4‑OHT (Bio‑4‑OHT) or excess unlabelled 4‑OHT (100 µM) incubated with A549 cell lysates. Complexes were captured with streptavidin beads and analysed by western blotting. (G) Surface plasmon resonance (SPR) analysis showing the binding affinity of 4‑OHT for AKR1B10. (H) Western blotting analysis of AKR1B10 expression in LUAD cells treated with 4‑OHT for 48 h. Vinculin served as a loading control. Representative blots from three independent experiments are shown. (I) Effects of Epalrestat (Epa) and 4‑OHT on AKR1B10 enzymatic activity (mean ± SD, *n* = 3, ns, not significant, *p* > .05; *p < .05, two‑way ANOVA with Tukey's post hoc test). (J) Western blotting analysis of AKR1B10 and GPX4 expression in H1299 cells transfected with control shRNA (sh#Ctrl) or AKR1B10 shRNAs (sh#1, sh#2). Vinculin served as a loading control. Representative blots from three independent experiments are shown. (K) Western blot analysis of 4‑HNE adduct levels in H1299 cells transfected with control shRNA or AKR1B10 shRNAs. Vinculin served as a loading control. Representative blots from three independent experiments are shown. (L) Relative lipid peroxidation levels in H1299 cells transfected with control shRNA or AKR1B10 shRNAs, measured by flow cytometry. Quantification of mean fluorescence intensity is shown (mean ± SD, *n* = 3, ***p < .001, one‑way ANOVA with Dunnett's post hoc test). (M) Free fatty acid (FFA) content in H1299 cells transfected with control shRNA or AKR1B10 shRNAs, measured by absorbance at 570 nm (mean ± SD, *n* = 3, **p < .01; ***p < .001, one‑way ANOVA with Dunnett's post hoc test). (N) GSH/GSSG ratio in H1299 cells transfected with control shRNA or AKR1B10 shRNAs (mean ± SD, *n* = 3, ***p < .001, one‑way ANOVA with Dunnett's post hoc test). (O) Western blotting analysis of AKR1B10 and GPX4 expression in AKR1B10‑knockout and AKR1B10‑rescue H1299 cells. Vinculin served as a loading control. Representative blots from three independent experiments are shown. (P) Cell death in control, AKR1B10‑knockout, and AKR1B10‑rescue H1299 cells with or without 4‑OHT treatment for 48 h, quantified by PI staining and flow cytometry (mean ± SD, *n* = 3, ns, not significant, *p* > .05; ***p < .001, two‑way ANOVA with Tukey's post hoc test). (Q) Cell viability of control, AKR1B10‑knockout, and AKR1B10‑rescue H1299 cells treated with 4‑OHT for 72 h, assessed by CCK‑8 assay. IC_50_ values were calculated by nonlinear regression (mean ± SD, *n* = 3, two‑way ANOVA). (R) Representative images of subcutaneous xenograft tumours from BALB/c nude mice inoculated with sg#NC, sg#AKR1B10, oe‑NC, or oe‑AKR1B10 H1299 cells (5 × 10^6^ cells), followed by DMSO or 4‑OHT (60 mg/kg) treatment (*n* = 5). (S) Quantification of xenograft tumour weights (mean ± SD, *n* = 5, ns, not significant, *p* > .05; ***p < .001, one‑way ANOVA with Tukey's post hoc test). (T) Immunohistochemical (IHC) staining of a LUAD tissue microarray with anti‑AKR1B10 antibody. N: adjacent normal tissue; T: tumour tissue. (U) Quantification of the IHC staining. (mean ± SD, *n* = 90, *** *p* < .001, unpaired Student's *t*‐test). (V) Kaplan–Meier survival curve of LUAD patients (*n* = 180) stratified by AKR1B10 expression (high: *n* = 90; low: *n* = 90). Statistical significance was determined by the log‑rank (Mantel–Cox) test.

LiP‑MS was performed on LUAD cells to identify candidate 4‑OHT‑binding proteins (Figure 6A; Tables S3 and S4). Among the candidate proteins, the Aldo‐keto reductase‐AKR1B10, which catalyses the reduction of carbonyl‐containing compounds to their corresponding alcohols, caught our attention (Figure 6B). Molecular docking demonstrated a strong binding between 4‐OHT and AKR1B10 with the binding energy of –9.3 kcal/mol, and 4‐OHT formed hydrogen bonds with Tyr49 and His111, which are located in the catalytic channel of AKR1B10 (Figure 6C and D).^37^ Subsequently, we connected Biotin to 4‐OHT molecule to synthesise Bio‐4‐OHT and pull‐down assays were performed in A549 cell lysate using streptavidin magnetic beads (Figure 6E). A competition group with excess non‑biotinylated 4‑OHT was included to verify binding specificity. Figure 6F showed that 4‐OHT interacted with AKR1B10, and Y49A/H111A double mutants almost completely eliminated this interaction (Figure S7A). To further confirm this binding, Surface Plasmon Resonance (SPR) technique was used, and the dissociation constant K_D_(M) was determined to be 7.98e‐06 (Figure 6G). Cellular thermal shift assay (CETSA) also showed that 4‐OHT treatment stabilised AKR1B10, indicating the intracellular binding of 4‐OHT to AKR1B10 (Figure S7B and C). Collectively, these results demonstrate that 4‑OHT directly binds to AKR1B10.

We next investigated how 4‑OHT influences AKR1B10 in LUAD cells. The protein levels of AKR1B10 were detected in LUAD cells treated with different concentrations of 4‐OHT. Figure 6H showed that 4‐OHT treatment did not apparently affect the expression of AKR1B10. We then tested the enzymatic activity of AKR1B10 treated with 4‐OHT or epalrestat, which has been demonstrated as an effective inhibitor of AKR1B10. The in vitro activity assay of AKR1B10 showed that 4‐OHT exhibited superior inhibitory potency against AKR1B10 compared to epalrestat (Figures 6I and S7D). We then knocked down AKR1B10 using specific shRNAs and found that the GSH/GSSG ratio, GPX4 expression, and intracellular free fatty acid levels were markedly decreased whereas lipid peroxidation and 4‐HNE adducts were increased (Figures 6J–N and S7E–G). To further confirm 4‐OHT‐induced cell death was mediated by AKR1B10, we constructed AKR1B10 knockout stable cell lines in H1299 (H1299‐sgAKR1B10) using CRISPR/Cas9 technique. The AKR1B10‐KO cell line showed a significantly reduced GSH/GSSG ratio, lower GPX4 expression, and decreased proliferation ability compared with control cell lines (Figure S7H–J). We treated this cell line with 4‐OHT and found that the cell death ratio was significantly decreased (Figure S7K–M). We also determined the IC_50_ of 4‐OHT in this cell line and Figure S7N showed that AKR1B10 knockout remarkably reduced the cell sensitivity to 4‐OHT. We reconstituted AKR1B10 expression in the sg‐AKR1B10 knockout H1299 cell line (AKR1B10‐rescue). We found that upon 4‐OHT treatment, the proportion of cell death was higher in the AKR1B10‐rescued group compared with the knockout group, and the cellular sensitivity to 4‐OHT was significantly restored (Figure 6O–Q).

We further validated the target function of AKR1B10 in vivo using a mouse xenograft model. Tumour volumes were significantly smaller in the sg‐AKR1B10 knockout group relative to the control group, whereas AKR1B10 overexpression partially accelerated tumour proliferation and reduced responsiveness to 4‐OHT. Collectively, these data further support that AKR1B10 serves as a target of 4‐OHT (Figures 6R and S and S7O).

Finally, AKR1B10 expression was then assessed in LUAD using a tissue microarray (Table S5). Immunohistochemical (IHC) analysis revealed a higher AKR1B10 expression in cancer tissues compared to adjacent normal tissues (Figure 6T and U), and the elevated AKR1B10 expression correlated with poor patient prognosis (Figure 6V). Meanwhile, bioinformatic survival analyses showed that elevated AKR1B10 expression correlates with unfavourable overall survival in lung cancer (Figure S7P). Notably, high AKR1B10 expression predicts worse survival outcomes among patients with chemotherapy‐resistant lung cancer (Figure S7Q). Moreover, AKR1B10 expression exhibited a significant positive correlation with chemotherapy resistance‐related genes including TUBB3, RRM1 and TYMS (Figure S7R–T).^38^, ^39^ These findings demonstrate that 4‐OHT induces a noncanonical ferroptosis by targeting AKR1B10.

We then focused on how AKR1B10 inhibition induces cell death. ACC1 is the first key enzyme in fatty acid synthesis that catalyses the conversion of acetyl‐CoA to malonyl‐CoA. Previous studies demonstrated that AKR1B10 promoted fatty acid synthesis by inhibiting the acetyl‐CoA carboxylase 1 (ACC1) degradation.^40^, ^41^, ^42^ In H1299 cells, we also found that either 4‐OHT treatment or AKR1B10 knockdown reduced the protein expression of ACC1 (Figure S8A and B), indicating that the lipid deficiency in the present cell death was partly caused by the reduced expression of ACC1. We also found that ACC1 mRNA was not altered by 4‐OHT (Figure S8C); CHX chase assays revealed that 4‐OHT accelerated ACC1 degradation, which was blocked by MG132 (Figure S8D), indicating proteasome‐dependent degradation. Notably, AKR1B10 knockout alone phenocopied the 4‐OHT‐induced ACC1 degradation. Collectively, these data demonstrate that AKR1B10 maintains ACC1 stability by preventing its proteasomal degradation. For lipid peroxidation, AKR1B10 was known to effectively reduce the RCSs, such as 4‐HNE and MDA generated from the cellular oxidation of lipids.^43^ From our results in Figures 4O–Q, 6K, and S6A–E, inhibiting AKR1B10 induced the accumulation of RCSs derived from lipid peroxidation. Therefore, the accumulation of RCSs is caused by the reduced enzymatic activity of AKR1B10.

### 3.7 Nanatinostat also induces noncanonical ferroptosis by targeting AKR1B10

**FIGURE 7:**
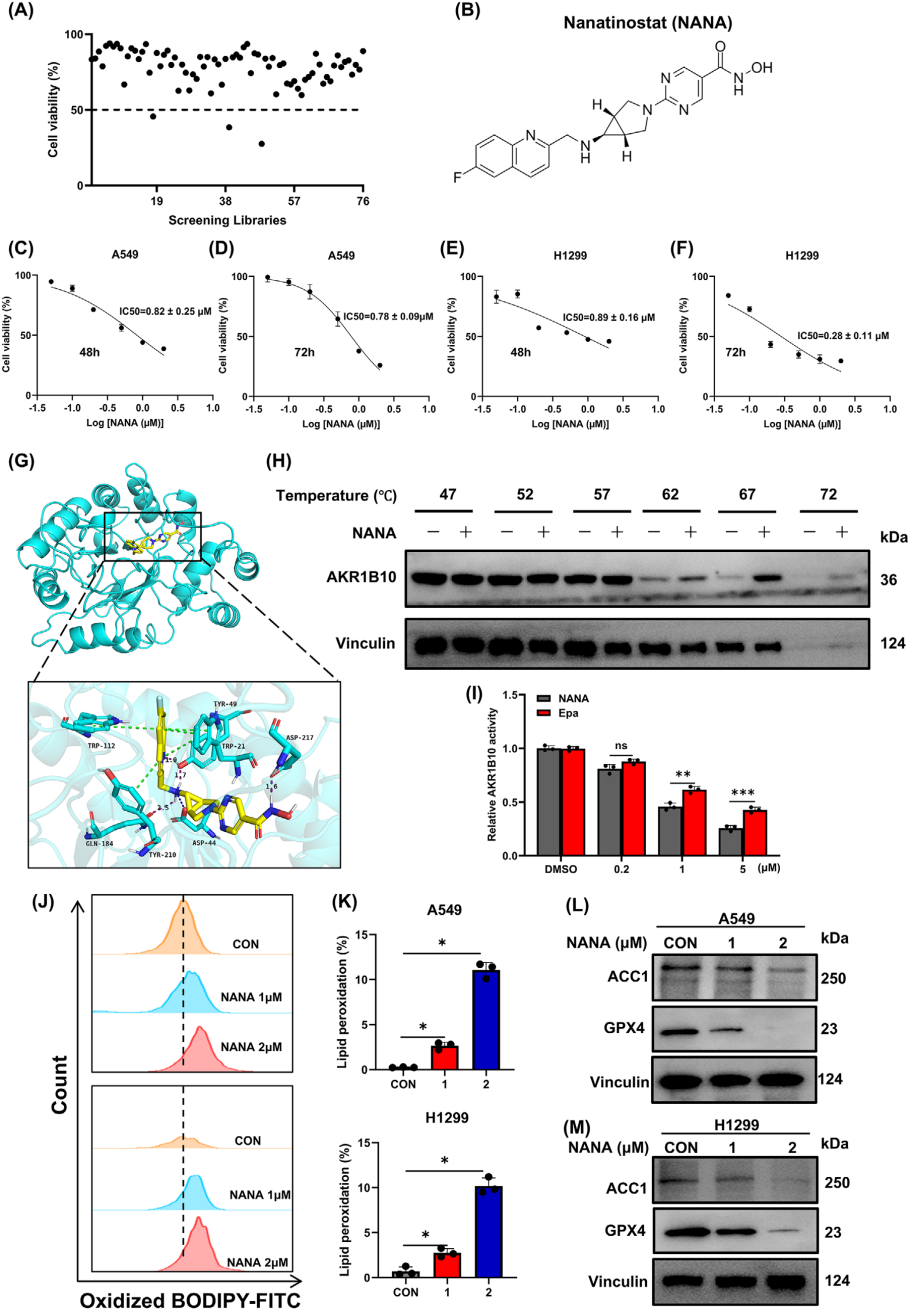
Nanatinostat also induces noncanonical ferroptosis by targeting AKR1B10. (A) High‐throughput virtual screening of drug repurposing and natural product libraries (total 12497 compounds) in A549 cells. Cell viability was assessed by CCK‐8 assay (10 µM, 24 h). The red circle represents Nanatinostat. (B) The chemical structure of Nanatinostat (NANA). (C–F) Dose–response curves of A549 (C and D) and H1299 (E and F) cells treated with Nanatinostat at indicated concentrations for 48 h or 72 h. Cell viability was assessed by CCK‐8 assay. IC_50_ values were calculated by nonlinear regression (mean ± SD, *n* = 3, two‐way ANOVA). (G) Molecular docking simulation of the binding between Nanatinostat and AKR1B10. (H) Cellular Thermal Shift Assay (CETSA) in A549 cells treated with Nanatinostat (10 µM) for 24 h, followed by western blotting. Representative blots from three independent experiments are shown. (I) Effects of Epalrestat (Epa) and Nanatinostat on AKR1B10 enzymatic activity (mean ± SD, *n* = 3, ns, not significant, *p* > .05; **p < .01; ***p < .001, two‑way ANOVA with Tukey's post hoc test). (J and K) Lipid peroxidation levels in A549 (J) and H1299 (K) cells treated with Nanatinostat (1 or 2 µM) for 48 h, measured by flow cytometry (mean ± SD, *n* = 3, *p < .05, one‑way ANOVA with Dunnett's post hoc test). (L and M) Western blot analysis of ACC1 and GPX4 expression in A549 (L) and H1299 (M) cells treated with Nanatinostat (1 or 2 µM) for 48 h. Vinculin served as a loading control. Representative blots from three independent experiments are shown.

To identify whether other drugs or compounds induce noncanonical ferroptosis by targeting AKR1B10, we conducted High‐Throughput Virtual Screening using AKR1B10 as the target protein in a Drug Repurposing Compound Library (6242 compounds) and a Natural Product Library (6255 compounds). The Top 200 compounds in each selected Library are shown in Tables S6 and S7, and we selected the top 50 compounds from each of the two libraries (excluding some duplicate compounds, a total of 76 compounds) for cell viability testing. Figure 7A showed that some compounds effectively inhibited the viability of A549 cells, among which Nanatinostat (NANA) exhibited the most potent inhibitory activity (Figure 7B). NANA is a class I‐selective histone deacetylase inhibitor, though its role in lung cancer remains largely unexplored. We found that NANA effectively inhibited the viability of LUAD cells with the IC_50_ below 1 µM (Figure 7C–F). Molecular docking showed that NANA formed hydrogen bonds with AKR1B10 at Tyr49, Gln184 and Asp217 (Figure 7G). CETSA showed that NANA treatment stabilised AKR1B10, indicating the intracellular binding of NANA to AKR1B10 (Figures 7H and S9A). The in vitro activity assay of AKR1B10 showed that NANA effectively inhibited its enzymatic activity and this inhibitory effect was superior to epalrestat (Figure 7I). Rescue experiments using specific inhibitors targeting the classical forms of programmed cell death revealed that none of these inhibitors consistently rescued NANA‐induced cell death except for palmitic acid (PA) (Figure S9B and C). Then we detected the lipid peroxidation level and Figure 7J and K demonstrated that NANA treatment significantly increased lipid peroxidation both in A549 and H1299 cells. NANA treatment also reduced ACC1 and GPX4 expression in a concentration‐dependent manner (Figures 7L and M and S9D–G). Collectively, these results demonstrate that Nanatinostat also induces a noncanonical ferroptosis by targeting AKR1B10.

### 3.8 4‐OHT synergises with RCSs‑generating chemotherapy drugs

**FIGURE 8:**
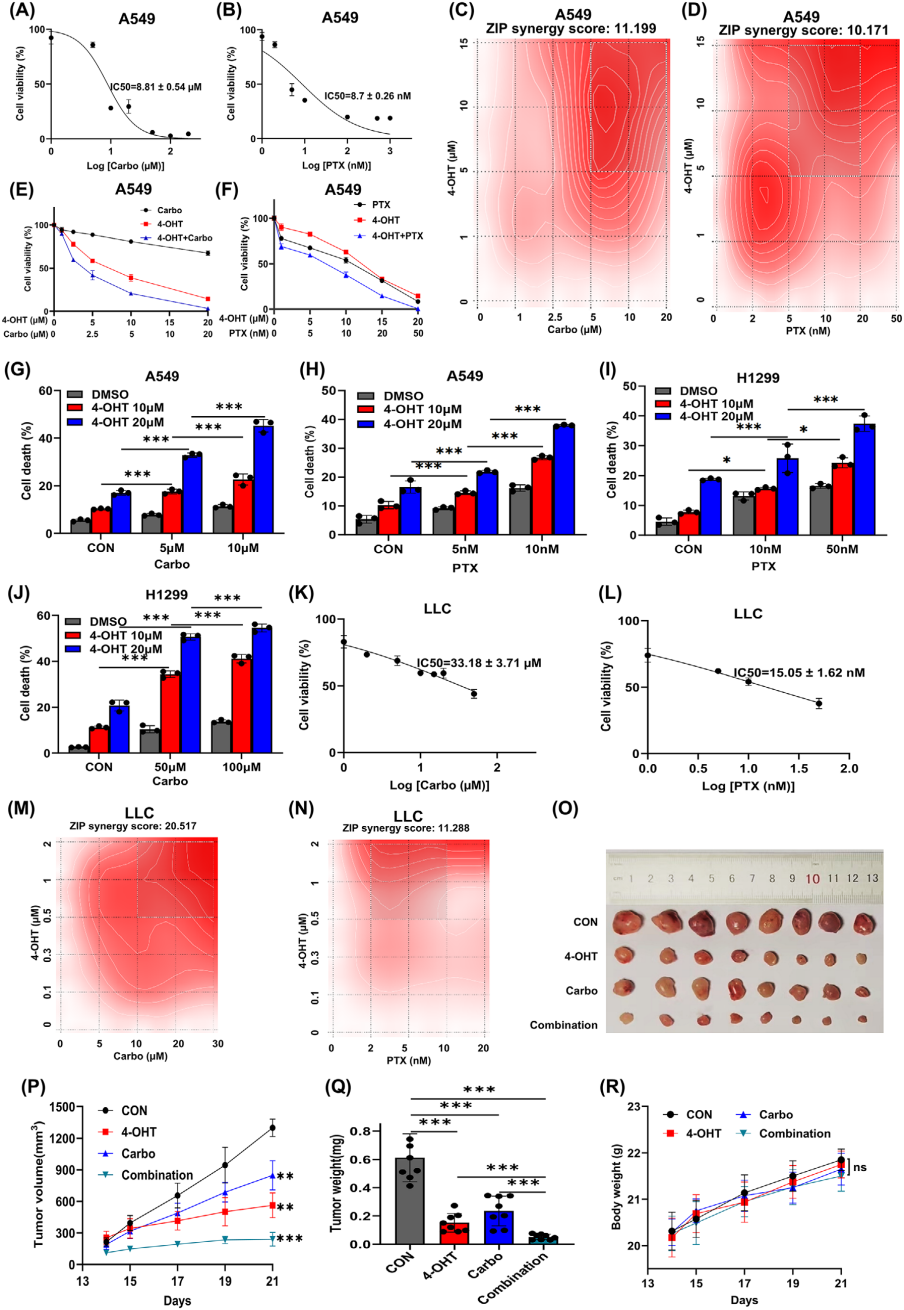
4‐OHT synergises with RCSs‑generating chemotherapy drugs. (A and B) Dose–response curves of A549 cells treated with carboplatin (A) or paclitaxel (PTX) (B) for 72 h. Cell viability was assessed by CCK‐8 assay. IC_50_ values were calculated by nonlinear regression (mean ± SD, *n* = 3, two‐way ANOVA). (C and D) Synergy analysis of 4‐OHT combined with carboplatin (C) or paclitaxel (D) in A549 cells for 72 h. Synergy scores were calculated using the zero‑interaction potency (ZIP) model. (E and F) Combination index analysis of A549 cells treated with fixed concentrations of 4‐OHT alone or in combination with carboplatin (E) or paclitaxel (F). Cell viability was measured by CCK‐8 assay (mean ± SD, *n* = 3). (G and H) Quantification of cell death in A549 cells treated with 4‐OHT (10 or 20 µM) alone or in combination with carboplatin (G) or paclitaxel (H) for 72 h. Cell death was assessed by PI staining and flow cytometry (mean ± SD, *n* = 3, ***p < .001, two‑way ANOVA with Tukey's post hoc test). (I and J) Quantification of cell death in H1299 cells treated with 4‐OHT (10 or 20 µM) alone or in combination with paclitaxel (I) or carboplatin (J) for 72 h. Cell death was assessed by PI staining and flow cytometry (mean ± SD, *n* = 3, *p  <  .05, ***p < .001, two‑way ANOVA with Tukey's post hoc test). (K and L) Dose–response curves of LLC cells treated with carboplatin (K) or paclitaxel (L) for 72 h. Cell viability was assessed by CCK‐8 assay. IC_50_ values were calculated by nonlinear regression (mean ± SD, *n* = 3, two‐way ANOVA). (M and N) Synergy analysis of 4‐OHT combined with carboplatin (M) or paclitaxel (N) in LLC cells. ZIP synergy scores were calculated as described. (O) Schematic diagram of the in vivo combination study. C57BL/6J mice engrafted with LLC cells received intraperitoneal administration of 4‐OHT (60 mg/kg), carboplatin (Carbo) (20 mg/kg), or combination for 7 days (*n* = 8 per group). (P) Tumour volume curves over time (mean ± SD, *n* = 8, **p < .01; ***p < .001, two‑way ANOVA with Tukey's post hoc test). (Q) Tumour weights at endpoint (mean ± SD, *n* = 8, ***p < .001, one‑way ANOVA with Tukey's post hoc test). (R) Body weight of the mice over time (mean ± SD, *n* = 8, ns, not significant, *p* > .05, two‑way ANOVA).

AKR1B10 has been reported to promote chemoresistance in cancer cells by reducing the specific carbonyl groups essential for the cytotoxicity of certain chemotherapeutic drugs, or the cytotoxic RCSs generation (e.g., carboplatin and paclitaxel).^43^, ^44^ We wanted to explore whether 4‐OHT could synergistically enhance the anticancer effect of chemotherapeutics that produce cytotoxic RCSs. In LUAD cells, both carboplatin and paclitaxel effectively inhibited the cell viability (Figures 8A and B and S10A and B), and 4‐OHT synergistically enhanced the efficacy of carboplatin and paclitaxel (Figures 8C–F and S10C and D). The combination index (CI) values calculated ranged from 0.45 to 0.78, indicating moderate to strong synergy (Figure S10E–J). Co‐treatment with 4‐OHT significantly potentiated the cell death induced by both agents (Figure 8G–J). Given that subsequent in vivo experiments were performed using the LLC syngeneic model, we first validated the synergistic effects in LLC cells.

Both chemotherapy drugs showed inhibitory effects with paclitaxel exhibiting a better anticancer efficacy (Figure 8K and L). The synergistic antitumour effects of 4‐OHT with carboplatin and PTX were further validated in LLC cells, and 4‐OHT showed potent synergistic anti‐cancer effects with each of the two drugs, especially with carboplatin (Figures 8M and N). The synergistic effect of 4‑OHT and carboplatin was further confirmed in C57BL/6J mice bearing LLC xenografts. The combination of 4‐OHT and carboplatin significantly enhanced the anti‐tumour effect compared to monotherapy, without adversely affecting mouse body weight (Figure 8O–R). Collectively, these findings suggest that 4‑OHT synergises with RCSs‑generating chemotherapy drugs.

## 4 DISCUSSION

Lung cancer is the leading cause of cancer death worldwide, with LUAD as its most prevalent subtype, comprising about 40% of cases.^1^, ^4^ Despite a decade of progress in targeted and immunotherapies, the 5‑year survival rate for LUAD patients has plateaued at approximately 20%–25%.^45^ A major clinical bottleneck is the widespread emergence of intrinsic and acquired drug resistance, including EGFR‐TKI resistance such as osimertinib failure, underscoring an urgent demand for repurposed small‐molecule therapies with novel mechanisms.^11^, ^46^, ^47^ In our study, we demonstrated that 4‐OHT induced a noncanonical ferroptosis not only in wild‐type LUAD cells but also in osimertinib‐resistant cell lines (PC9‐OR and HCC827‐OR). Notably, while epalrestat remains the only approved AKR1B10 inhibitor to date, our work identifies two novel AKR1B10 inhibitors: 4‐OHT and Nanatinostat, both exhibiting superior inhibitory effects compared to epalrestat.

Classical ferroptosis was initially defined as an iron‐dependent lipid peroxidation driven by disrupted GSH‐GPX4 signalling; however, growing contemporary research has broadened this framework to encompass noncanonical ferroptosis.^15^, ^48^ The cell death modality characterised in the present study retains canonical ferroptotic hallmarks, including GSH exhaustion, GPX4 loss, and lipid peroxidation. This mechanistic divergence is underscored by the failure of iron chelation, iron supplementation, or the classical ferroptosis inhibitor Fer‑1 to reverse 4‑OHT‑induced cell death. At the centre of this death program lies AKR1B10, which sustains LUAD cell survival via two metabolic axes: it stabilises ACC1 to support de novo fatty acid synthesis, while its reductase activity serves to neutralise cytotoxic lipid‑derived carbonyl species such as 4‑HNE.^41^, ^42^, ^49^, ^50^, ^51^, ^52^ Pharmacological inhibition of AKR1B10 by 4‐OHT appears to disrupt both protective pathways, precipitating concurrent lipid deficiency and toxic carbonyl accumulation that collectively initiate noncanonical ferroptosis, pending further lipidomic analysis.

Regarding the failure of Fer‑1 to rescue 4‑OHT‑induced cell death, Fer‑1 interrupts iron‑driven lipid peroxyl radical chain reactions in canonical ferroptosis.^53^, ^54^ However, AKR1B10 inhibition appears to generate cytotoxicity primarily through electrophilic aldehydes (e.g., 4‑HNE) rather than through radical propagation, rendering Fer‑1 ineffective.^55^, ^56^, ^57^ Notably, DFO, classically used as an iron chelator to suppress canonical ferroptosis, paradoxically enhanced 4‑OHT‑induced cell death, possibly via iron‑independent effects such as HIF‑1α stabilisation or mitochondrial stress, an intriguing direction for future investigation.^58^

Our mechanistic dissection further reveals that 4‑OHT treatment reduces the protein abundance of both ACC1 and GPX4 through distinct post‑translational regulatory cascades. The functional linkage between AKR1B10 and ACC1 has been well established in prior genetic investigations. AKR1B10 depletion triggers ubiquitin‑proteasome‑mediated ACC1 degradation, with COP1 identified as the responsible E3 ligase.^40^, ^41^ Whereas earlier investigations relied exclusively on genetic manipulation, our work provides complementary pharmacological evidence that small‑molecule inhibition of AKR1B10 recapitulates ACC1 destabilisation.

Regarding the mechanism linking AKR1B10 inhibition to GPX4 downregulation, our CHX assays confirmed that 4‑OHT accelerates GPX4 degradation via the proteasome. However, the upstream mechanism remains unclear. Based on published evidence that 4‑HNE triggers K48‑linked polyubiquitination and subsequent proteasomal degradation of GPX4 by disrupting its interaction with the deubiquitinase OTUD5.^55^, ^59^, ^60^, ^61^ We speculate that accumulated carbonyl stress may promote GPX4 turnover, while GSH depletion may further destabilise the protein by removing its essential cofactor. Direct assays, such as ubiquitination detection and E3 ligase identification are needed to validate this hypothesis. Therefore, AKR1B10 appears to sustain GPX4 abundance primarily by limiting carbonyl stress rather than via direct interaction. This potential dual mechanism – carbonyl‑induced GPX4 destabilisation coupled with cofactor deprivation – not only distinguishes our findings from classical ferroptosis but also highlights AKR1B10 as a candidate node linking lipid metabolism and redox homeostasis in LUAD.

Our findings establish that 4‐OHT exerts potent anticancer effects in LUAD through a mechanism distinct from its canonical ER‐antagonistic role in breast cancer.^33^ Moreover, we identified a synergistic anti‐tumour effect in LUAD when 4‐OHT was combined with chemotherapy (paclitaxel/carboplatin). As the target of 4‐OHT in LUAD cells, AKR1B10 confers chemotherapy resistance through the following pathways: it stabilises acetyl‐CoA carboxylase 1 (ACC1) to sustain de novo lipogenesis, thereby maintaining membrane integrity and limiting intracellular PTX accumulation^39^, ^62^; simultaneously, it uses NADPH to detoxify lipid peroxidation‐derived aldehydes (e.g., 4‐HNE), attenuating oxidative damage and creating a self‐reinforcing resistance loop.^44^ Furthermore, the carbonyl groups essential for PTX cytotoxicity can be reduced by AKR1B10.^43^ Collectively, these functions position AKR1B10 as a protective shield for tumour cells against chemotherapy. The concept of pharmacologically inducing ferroptosis to overcome chemoresistance is further supported by recent reviews highlighting the therapeutic potential of ferroptosis induction in reversing drug resistance.^11^, ^63^ This model is further supported by TCGA transcriptomic correlation showing positive associations between AKR1B10 and established chemoresistance markers including TUBB3, TYMS, and RRM1. Chou–Talalay CI calculations and in vivo xenograft data consistently demonstrate combinatorial antitumour activity without overt systemic toxicity or weight loss, reinforcing AKR1B10 inhibition as a viable strategy to overcome paclitaxel and carboplatin‐based resistance in LUAD.^64^ Regarding Nanatinostat as an additional AKR1B10 inhibitor, we note that its IC_50_ values in LUAD cells are substantially lower than those of 4‑OHT. This likely reflects its greater on‑target potency rather than non‑specific off‑target effects, as Nanatinostat recapitulated the same core phenotypes‐including ACC1/GPX4 degradation and lipid peroxidation‐and rescue by palmitic acid (PA). Nevertheless, as a class I‑selective HDAC inhibitor, Nanatinostat may have additional targets; dissecting the relative contribution of AKR1B10 versus HDAC inhibition will require further investigation using AKR1B10‑specific probes or isoform‑selective analogues. Recent studies on ferroptosis‑associated gene signatures in tumour prognosis and microenvironment remodelling provide valuable methodological frameworks that further support the translational relevance of our findings.^17^ Moreover, emerging evidence on the crosstalk between ferroptosis and immune signalling pathways, such as cGAS‑STING, offers new perspectives for combining ferroptosis‑inducing strategies with immunotherapy in future translational studies.^65^, ^66^, ^67^, ^68^

This study has several limitations that warrant consideration. First, while we confirmed specific binding and enzymatic inhibition of AKR1B10 by both 4‑OHT and Nanatinostat, comprehensive selectivity profiling against the closely homologous AKR1B1 isoform remains to be performed, leaving potential off‑target contributions unresolved. Second, the iron‑independent nature of this cell death was validated only through in vitro assays; complementary in vivo models (e.g., low‑iron diet or tumour‑specific transferrin knockdown) are needed to confirm this phenotype within the tumour microenvironment. Third, although proteasomal GPX4 clearance was confirmed, the specific E3 ubiquitin ligase responsible for recognising carbonyl‑modified GPX4 has yet to be identified. Fourth, all mechanistic and therapeutic validation was performed using immortalised cell lines and murine xenografts, without complementary testing in patient‑derived organoids or retrospective clinical cohorts to establish AKR1B10 as a predictive biomarker for chemotherapy response. Fifth, we did not assess whether 4‑OHT alters carboplatin pharmacokinetics, and our in vivo combination studies used a single dosing schedule; therefore, dose optimisation may further improve the therapeutic window. Future work will address these gaps through isoform‐selective screening, iron‐manipulated in vivo models, E3 ligase identification, and translational validation using primary human specimens; additional investigations will explore combinatorial regimens with immune checkpoint inhibitors and assess this pathway in other AKR1B10‐high solid tumours.

## 5 CONCLUSIONS

This study indicates that 4‑OHT inhibits AKR1B10 enzymatic activity to induce a noncanonical ferroptosis in LUAD. Nanatinostat similarly targets AKR1B10 and elicits the same death modality, and AKR1B10 overexpression correlates with poor prognosis and chemoresistance in LUAD patients. These findings suggest AKR1B10 inhibition as a candidate therapeutic strategy, but the underlying mechanisms warrant further investigation.

## AUTHOR CONTRIBUTIONS

Hui Yang and Tianyu Han conceived and designed the study, carried out the experiments, analysed the data, and drafted the original manuscript. Yanan Wang and Jiangbo Jin contributed to the methodology and supervised data collection. Hui Yang, Hanyan Xu, and Yinshui Miao assisted with the experimental setup and performed data collection. Research oversight and guidance were provided by Xiaojing Wu, Shengsong Chen, Tianyu Han, and Qingyuan Zhan. All authors have reviewed and approved the final manuscript.

## ETHICS STATEMENT

Animal experimentation was conducted in accordance with the guidelines of the Laboratory of Animals, The first affiliated hospital of Nanchang University.

## CONFLICT OF INTEREST STATEMENT

The authors declare no conflicts of interest.

## Supporting information



Supporting Information

Supporting Information

Supporting Information

Supporting Information

Supporting Information

Supporting Information

Supporting Information

Supporting Information

Supporting Information

Supporting Information

## Data Availability

All data supporting this study are available from the corresponding authors on reasonable request.
