# Supplementary material for: 4‐Hydroxytamoxifen induces a noncanonical ferroptosis in lung adenocarcinoma by targeting AKR1B10
Source: Clin Transl Med. 2026 Sep 8;16(9):e70796. doi: 10.1002/ctm2.70796 (PMC13551091; doi:10.1002/ctm2.70796)
Supplement: Supplementary file 1 [file CTM2-16-e70796-s001.docx]

**Figure S1 4-OHT inhibits the viability of LUAD cells.**

**Figure S2 Quantification of cell cycle-related proteins upon 4-OHT treatment.**

**Figure S3** **Validation of cell death inhibitors and characterization of cell death pathways.**

**Figure S4 Validation of classical programmed cell death pathways in 4-OHT-induced cell death.**

**Figure S5 Palmitic acid effectively rescues cell death induced by 4-OHT.**

**Figure S6 4-OHT induces the increased production of 4-hydroxynonenal (4-HNE).**

**Figure S7** **4-OHT induces noncanonical ferroptosis by targeting AKR1B10.**

**Figure S8** **4-OHT induces noncanonical ferroptosis by targeting AKR1B10.**

**Figure S9 NANA also induces noncanonical ferroptosis by targeting AKR1B10.**

**Figure S10** **4-OHT synergizes with RCSs‑generating chemotherapy drugs.**

**Supplemental Tables**

**Table S1.** The detailed information of 950 human endogenous metabolic compounds.

**Table S2.** The results of the untargeted metabolomics.

**Table S3.** The identified characteristic sequences by LiP-MS (4-OHT high-dose group VS Control group).

**Table S4.** The identified characteristic sequences by LiP-MS (4-OHT low-dose group VS Control group).

**Table S5.** The detailed pathological data of LUAD tissue microarray.

**Table S6.** The Top200 compounds identified by High-Throughput Virtual Screening using the Natural Product Library.

**Table S7.** The Top200 compounds identified by High-Throughput Virtual Screening using the Drug Repurposing Compound Library.

**Table S8.** The resources table.
