# Supplementary material for: 4‐Hydroxytamoxifen induces a noncanonical ferroptosis in lung adenocarcinoma by targeting AKR1B10
Source: Clin Transl Med. 2026 Sep 8;16(9):e70796. doi: 10.1002/ctm2.70796 (PMC13551091; doi:10.1002/ctm2.70796)
Supplement: Supplementary file 2 [file CTM2-16-e70796-s002.docx]

**METHODS**

**• KEY RESOURCES TABLE**

| Antibodies | Source | Identifier |
| --- | --- | --- |
| Anti-CDK2 | ZenBio | Cat# R22532 |
| Anti-CDK4 | ZenBio | Cat# R23886 |
| Anti-CDK6 | ZenBio | Cat# R23891 |
| Anti-CyclinB1 | ZenBio | Cat# R23324 |
| Anti-CyclinD1 | ZenBio | Cat# R380999 |
| Anti-CyclinE1 | ZenBio | Cat# R381029 |
| Anti-SLC7A11 | ZenBio | Cat# R26116 |
| Anti-FSP1 | ZenBio | Cat#R342551 |
| Anti-ACSL4 | ABclonal | Cat# A20414 |
| Anti-GPX4 | ABclonal | Cat# A1933 |
| Anti-Caspase-1 | ABclonal | Cat# A0964 |
| Anti-GSDME | ABclonal | Cat# A7432 |
| Anti-MLKL | ABclonal | Cat# A5579 |
| Anti-P-MLKL | ABclonal | Cat# AP1244 |
| Anti-AKR1B10 | ABclonal | Cat# A7823 |
| Anti-4-HNE | ABclonal | Cat# A26085 |
| Anti-NLRP3 | ABclonal | Cat# A24294 |
| Anti-CDK1 | Proteintech | Cat# 19532-1-AP |
| Anti-SQSTM1/p62 | Proteintech | Cat# 18420-1-AP |
| Anti-Vinculin | Proteintech | Cat# 26520-1-AP |
| Anti-LC3 | Proteintech | Cat# 14600-1-AP |
| Anti-Beclin1 | Proteintech | Cat# 11306-1-AP |
| Anti-H2AX | Proteintech | Cat# 10856-1-AP |
| Anti-r-H2AX | Proteintech | Cat# 29380-1-AP |
| Anti-Beclin1 | Proteintech | Cat# 11306-1-AP |
| Anti-ACC1 | Proteintech | Cat# 81656-1-RR |
| Anti-Caspase 3 | Proteintech | Cat# 19677-1-AP |
| Anti-Bcl-2 | Proteintech | Cat# 26593-1-AP |
| Anti-BAX | Proteintech | Cat# 50599-2-Ig |
| Anti-RIPK1 | Proteintech | Cat# 29932-1-AP |
| Anti-RIPK3 | Proteintech | Cat# 29080-1-AP |
| Anti-DYKDDDDK-tag | Proteintech | Cat# 66008-4-Ig |
| Anti-Estrogen Related Receptor α | Selleckchem | Cat# F1190 |
| Anti-Estrogen Related Receptor β | Selleckchem | Cat# A5691 |
| Anti-GSDMD | Selleckchem | Cat# F1568 |
| Anti-cleaved-Caspase 3 | Selleckchem | Cat# F0135 |
|  |  |  |
| Reagents | **Source** | **Identifier** |
| (E/Z)-4-Hydroxytamoxifen | MedChemExpress | Cat# HY-16950A |
| Nanatinostat | MedChemExpress | Cat# HY-13432 |
| DMSO | Sigma | Cat# C6628 |
| TCEP Hydrochloride | Selleckchem | Cat# S4611 |
| DL-Glyceraldehyde | Orileaf | Cat# S30596 |
| 3-Methyladenine (3-MA) | MedChemExpress | Cat# HY-19312 |
| NH4CL | MedChemExpress | Cat# HY-Y1269 |
| Ferrostatin-1 | MedChemExpress | Cat# HY-100579 |
| Necrostatin-1 | MedChemExpress | Cat# HY-15760 |
| Trolox | MedChemExpress | Cat# HY-101445 |
| Z-VAD-FMK | MedChemExpress | Cat# HY-16658B |
| PMSF | MedChemExpress | Cat# HY-B0496 |
| N-Acetylcysteine (NAC) | MedChemExpress | Cat# HY-B0215 |
| Necrosulfonamide (NSA) | MedChemExpress | Cat# HY-100573 |
| DCFH-DA | MedChemExpress | Cat# HY-D0940 |
| Palmitic acid (PA) | MedChemExpress | Cat# HY-N0830 |
| Deferoxamine (DFO) | MedChemExpress | Cat# HY-B1625 |
| Erastin | MedChemExpress | Cat# HY-15763 |
| RSL3 | MedChemExpress | Cat# HY-100218A |
| Epalrestat | MedChemExpress | Cat# HY-66009 |
| 4-Hydroxynonenal | MedChemExpress | Cat# HY-113466 |
| Paclitaxel | MedChemExpress | Cat# HY-B0015 |
| Carboplatin | MedChemExpress | Cat# HY-17393 |
| PEG300 | MedChemExpress | Cat# HY-Y0873 |
| Tween 80 | MedChemExpress | Cat# HY-Y1891 |
| Fulvestrant | MedChemExpress | Cat# HY-13636 |
| Streptavidin Magnetic Beads | MedChemExpress | Cat# HY-K0208 |
| Propidium Iodide | MedChemExpress | Cat# HY-D0815 |
| Puromycin | MedChemExpress | Cat# HY-K1057 |
| Osimertinib | MedChemExpress | Cat# HY-15772 |
| RIPA Lysis Buffer | Beyotime | Cat# P0013B |
| NADPH | Beyotime | Cat# ST360 |
| Phosphate Buffered Saline (PBS) | Servicebio | Cat# G4202-500ML |
| non-fat milk | Solarbio | Cat# D8340 |
| Fetal Bovine Serum | Excell | Cat# FSD500 |
| Cycloheximide | MedChemExpress | Cat# HY-12320 |
| TNF-α | MedChemExpress | Cat# HY-P1860 |
| MG132 | MedChemExpress | Cat# HY-13259 |
| Ferric ammonium citrate (FAC) | MedChemExpress | Cat# HY-B1645 |
| Necroptosis Inducer Kit with TSZ | Beyotime | Cat# C1058S |
| Perchloric acid | Aladdin | Cat# P112070-500ml |
| TRIzol | Solarbio | Cat# 19201ES60 |
| Matrigel Matrix | Corning | Cat# 354262 |
| Experimental models: Cell lines | | |
| HEK-293T | Gift from others | N/A |
| MCF-7 | National Collection of Authenticated Cell Cultures | Cat#[TCHu 74](https://cellbank.org.cn/search-detail.php?id=152) |
| A549 | National Collection of Authenticated Cell Cultures | Cat#TCHu150 |
| H1299 | National Collection of Authenticated Cell Cultures | Cat#TCHu160 |
| HCC827 | Gift from others | N/A |
| PC9 | Gift from others | N/A |
| PC9-OR | Gift from others | N/A |
| HCC827-OR | Gift from others | N/A |
| LLC | ubigene | Cat#YC-C018-Luc-P |
| The primers used for PCR |  |  |
| *GPX4-F* | TGTAACCAGTTCGGGAAGCA | |
| *GPX4-R* | TCCACTTGATGGCATTTCCC | |
| *ACC1-F* | TGGTGCAGAGGTACCGAAGTG | |
| *ACC1-R* | CTGCGGCAGCAGATCCAT | |
| *GAPDH-F* | GAAGGTGAAGGTCGGAGTC | |
| *GAPDH-R* | GAAGATGGTGATGGGATTTC | |
| Experimental models: Organisms/strains | | |
| Mouse: C57BL/6J  BALB/c-Nude | Jicui Pharmachem | N/A |
| Critical commercial assays | | |
| Cell Cycle and Apoptosis Analysis Kit | Yeasen | Cat#40301ES50 |
| Amplex Red Free Fatty Acid Assay Kit | Beyotime | Cat# S0215S |
| Cell Counting Kit-8 | Abmole | Cat# M4839 |
| Enhanced ECL Chemiluminescent Substrate Kit | Yeasen | Cat# 36222ES60 |
| Annexin V-FITC/PI Apoptosis Kit | Elabscience | Cat# E-CK-A211 |
| Cytotoxicity LDH Assay Kit | MedChemExpress | Cat# HY-K1090 |
| Lipid Peroxidation MDA Assay Kit | Beyotime | Cat# S0131S |
| Enhanced BCA Protein Assay Kit | Beyotime | Cat# P0010S |
| Phen Green SK diacetate | MedChemExpress | Cat# HY-126823 |
| Lipid Peroxidation Assay Kit with BODIPY 581/591 C11 | Beyotime | Cat# S0043S |
| GSH and GSSG Assay Kit | Beyotime | Cat# S0053 |
| jetPRIMER® transfection reagent | Polyplus | Cat# 101000046 |
| Software and algorithms | | |
| GraphPad prism 10 | GraphPad | N/A |
| FlowJo 10 | BD Biosciences | N/A |
| AutoDock 4 | Scripps | N/A |
| LigPlot 2 | EMBL-EBI | N/A |
