# Supplementary material for: 4‐Hydroxytamoxifen induces a noncanonical ferroptosis in lung adenocarcinoma by targeting AKR1B10
Source: Clin Transl Med. 2026 Sep 8;16(9):e70796. doi: 10.1002/ctm2.70796 (PMC13551091; doi:10.1002/ctm2.70796)
Supplement: Supplementary file 3 [file CTM2-16-e70796-s004.docx]

**Figure S1 4-OHT inhibits the viability of LUAD cells.**

(A-D) Validation of osimertinib‑resistant (OR) cell lines. PC9 (A), PC9‑OR (B), HCC827 (C), and HCC827‑OR (D) cells were treated with increasing concentrations of osimertinib for 72 h. Cell viability was assessed by CCK‑8 assay. IC_50_ values were calculated by nonlinear regression (mean ± SD, n = 3, two-way ANOVA). (E-H) PC9 (E), PC9‑OR (F), HCC827 (G), and HCC827‑OR (H) cells were treated with 4-OHT, and cell proliferation was detected at the indicated time. Cell growth was assessed using crystal violet staining, and the data represent the mean values of three independent experiments (mean ± SD, n = 3, ****P* < 0.001, two‑way ANOVA with Tukey‘s post‑hoc test). (I) Colony formation assay in LUAD cell lines (A549, H1299, PC9, PC9‑OR, HCC827, and HCC827‑OR) treated with 4‑OHT for 10 days. Representative images from three independent experiments are shown. (J) Cell viability of LLC cells treated with 4-OHT at various concentrations for 72h, assessed by CCK-8 assays. IC_50_ values were calculated by nonlinear regression (mean ± SD, n = 3, two-way ANOVA). (K) LLC cells were treated with 4-OHT, and cell proliferation was assessed by crystal violet staining at the indicated time points (mean ± SD, n = 3, ****P*  <  0.001, two‑way ANOVA with Tukey‘s post‑hoc test). (L) Colony formation assay in LLC cells treated with 4-OHT for 10 days. Representative images from three independent experiments are shown. (M) Body weights of the allograft mice measured at indicated time points (mean ± SD, n = 7, ns, not significant, *P* > 0.05, two‑way ANOVA). (N) Western blot analysis of ERα and ERβ expression in LUAD cell lines. Vinculin served as a loading control. Representative blots from three independent experiments are shown. (O and P) Western blot analysis of ERα (O) and ERβ (P) expression in A549 cells with Erα/ERβ knockout. Vinculin served as a loading control. Representative blots from three independent experiments are shown.

**Figure S2 Quantification of cell cycle-related proteins upon 4-OHT treatment.**

(A–G) Quantitative analysis of relative protein levels of CDK1, CDK2, CDK4, CDK6, cyclin B1, cyclin D, and cyclin E1 in LUAD cell lines (A549, H1299, HCC827, HCC827‑OR, PC9, and PC9‑OR) treated with 4‑OHT (0, 10, 15, or 20 μM). Band intensities were normalized to Vinculin (mean ± SD, n = 3, ns, not significant, *P* > 0.05, two‑tailed unpaired Student‘s t‑test). (H–N) Relative protein levels of CDK1, CDK2, CDK4, CDK6, Cyclin B1, Cyclin D and Cyclin E1 in MCF-7 cells treated with 4-OHT (0, 10, or 15 μM). Band intensities were normalized to Vinculin (mean ± SD, n = 3, ***P* < 0.01, ****P* < 0.001, two‑tailed unpaired Student‘s t‑test).

**Figure S3** **Validation of cell death inhibitors and characterization of cell death pathways.** (A) Apoptosis validation. H1299 cells were treated with TNF‑α+ cycloheximide (CHX) alone or in combination with Z‑VAD‑FMK for 24 h. Cell viability was assessed by CCK‑8 assay (mean ± SD, n = 3, ****P* < 0.001, two‑tailed unpaired Student's t‑test). (B) Necroptosis validation. HT‑29 cells were treated with TSZ (TNF‑α + SM‑164 + Z‑VAD‑FMK) alone or in combination with necrostatin‑1 (Nec‑1) for 24 h. Cell viability was assessed by CCK‑8 assay (mean ± SD, n = 3, ****P* < 0.001, two‑tailed unpaired Student's t‑test). (C) Necroptosis validation. H1299 cells were treated with TSZ (TNF‑α + SM‑164 + Z‑VAD‑FMK) alone or in combination with necrosulfonamide (NSA) for 24 h. Cell viability was assessed by CCK‑8 assay (mean ± SD, n = 3, ****P*  <  0.001, two‑tailed unpaired Student's t‑test). (D) Ferroptosis validation. H1299 cells were treated with RSL3 alone or in combination with ferrostatin‑1 (Fer-1) for 24 h. Cell viability was assessed by CCK‑8 assay (mean ± SD, n = 3, ****P*  <  0.001, two‑tailed unpaired Student's t‑test). (E) H1299 cells were treated with Erastin alone or in combination with NAC for 24 h. Cell viability was assessed by CCK‑8 assay (mean ± SD, n = 3, ****P*  <  0.001, two‑tailed unpaired Student's t‑test). (F) Autophagy validation. H1299 cells were treated with 3‑methyladenine (3‑MA, 5 mM) for 24 h. LC3‑I and LC3‑II levels were detected by Western blotting. Vinculin served as a loading control. Representative blots from three independent experiments are shown. (G) ROS scavenger validation. H1299 cells were pretreated with Trolox for 1 h followed by H₂O₂ for 3 h. Intracellular ROS levels were measured using DCFH‑DA fluorescence (mean ± SD, n = 3, ****P*  <  0.001, two‑tailed unpaired Student's t‑test). (H) Cell death in H1299 cells induced by 4‑OHT could not be rescued by various cell death inhibitors. Cell death was quantified by PI staining and flow cytometry (mean ± SD, n = 3, ns, not significant, *P* > 0.05, one‑way ANOVA with Tukey's post‑hoc test). (I‑L) PANoptosis inhibitor combinations fail to rescue 4‑OHT‑induced cell death. PC9 (I), PC9‑OR (J), HCC827 (K), and HCC827‑OR (L) cells were treated with the inhibitor Z‑VAD‑FMK, Nec‑1, NSA alone or in combination with 4‑OHT for 48 h. Cell viability was assessed by CCK‑8 assay (mean ± SD, n = 3, ****P*  <  0.001, two‑way ANOVA with Tukey‘s post‑hoc test). (M‑O) Quantification of FSP1(M), SLC7A11(N) and ACSL4(O), protein levels normalized to Vinculin (mean ± SD, n = 3, ns, not significant, *P* > 0.05, one‑way ANOVA with Tukey's post‑hoc test). (P) A549 cells were treated with DMSO or 4-OHT (10 or 20 μM) for 48 h. GAPDH served as the internal control (mean ± SD, n = 3, ns, not significant, *P* > 0.05, one‑way ANOVA with Dunnett's post‑hoc test). (Q) A549 cells were treated with cycloheximide (CHX, 50 μg/mL) alone, CHX plus 4-OHT (20 μM), or CHX plus 4-OHT (20 μM) and MG132 (10 μM) for the indicated time points. GPX4 protein levels were analyzed by western blotting. Vinculin served as a loading control. Representative blots from three independent experiments are shown.

**Figure S4 Validation of classical programmed cell death pathways in 4-OHT-induced cell death.** (A) Western blot analysis of apoptosis-related proteins (Bcl-2, Bax, Cleaved-Caspase-3, Caspase-3) in LUAD cells treated with 4-OHT for 48 h. Vinculin served as a loading control. Representative blots from three independent experiments are shown. (B) Western blot analysis of necrosis-related proteins (MLKL, p-MLKL, RIPK3, RIPK1) in LUAD cells treated with 4-OHT for 48 h. Vinculin served as a loading control. Representative blots from three independent experiments are shown. (C) Western blot analysis of autophagy-related proteins (p62, Beclin1, LC3B) in LUAD cells treated with 4-OHT for 48 h. Representative blots from three independent experiments are shown. (D) Western blot analysis of pyroptosis‑related proteins (GSDMD and GSDME) in LUAD cells treated with 4‑OHT (10 or 20 μM) for 48 h. Asterisk (*) indicates the N‑terminal fragment of GSDMD (N‑GSDMD). Vinculin served as a loading control. Representative blots from three independent experiments are shown.

**Figure S5 Palmitic acid effectively rescues cell death induced by 4-OHT.** (A-J) LUAD cell lines were treated with 4‑OHT (20 μM) alone or in combination with PA (25 μM) for 48 h. Cell death was quantified by PI staining and flow cytometry (mean ± SD, n = 3, **P* < 0.05; ****P*  <  0.001, two‑way ANOVA with Tukey‘s post‑hoc test). (K) Quantification of 4-HNE protein levels normalized to Vinculin (mean ± SD, n = 3, ***P* < 0.01; ****P* < 0.001, two‑tailed unpaired Student‘s t‑test).

**Figure S6 4-OHT induces the increased production of 4-hydroxynonenal (4-HNE).**

(A-E) Intracellular 4-hydroxynonenal (4-HNE) levels in positive control (treated with 4‑OHT) (A), H1299 (B), A549 (C), HCC827 (D), and PC9 (E) cells were quantified by high-performance liquid chromatography (HPLC).

**Figure S7** **4-OHT induces noncanonical ferroptosis by targeting AKR1B10.** (A) Biotinylated 4-OHT was incubated with the lysates of A549 cells transfected with pCMV-Flag-AKR1B10-WT or the mutants (Y49A, H111A and Y49A/H111A double mutants). Complexes were captured using streptavidin magnetic beads, washed, and eluted then detected by western blotting. (B) A549 cells were treated with 4-OHT for 24 h and Cellular Thermal Shift assay (CETSA) was performed followed by western blotting. (C) Quantitative analysis of relative AKR1B10 protein levels derived from CETSA immunoblots. Band intensities were normalized to Vinculin (mean ± SD, n = 3, ****P* < 0.001, two‑tailed unpaired Student‘s t‑test). (D) Dose–response analysis of cell viability upon exposure to serial concentrations of Epalrestat (Epa) and 4-OHT. IC_50_ values were calculated by nonlinear regression (mean ± SD, n = 3, two-way ANOVA). (E-G) Quantification of AKR1B10 (E), GPX4 (F), and 4‑HNE (G) protein levels in H1299 cells transfected with control shRNA or AKR1B10 shRNAs. Band intensities were normalized to Vinculin (mean ± SD, n = 3, ***P* < 0.01; ****P* < 0.001, two‑tailed unpaired Student's t‑test). (H) Western blot analysis of AKR1B10 knockout efficiency and GPX4 expression in H1299 cells. Vinculin served as a loading control. Representative blots from three independent experiments are shown. (I) GSH/GSSG ratio in H1299 cells with or without AKR1B10 knockout (mean ± SD, n = 3, ****P* < 0.001, two‑tailed unpaired Student's t‑test). (J) Cell proliferation of H1299 cells with or without AKR1B10 knockout, assessed by crystal violet staining at the indicated time points (mean ± SD, n = 3, ****P* < 0.001, two‑way ANOVA with Tukey's post‑hoc test). (K) Representative images of H1299 cells with or without AKR1B10 knockout treated with 4‑OHT for 48 h. Scale bars: 500 μm. (L and M) Cell death in H1299 cells with or without AKR1B10 knockout treated with 4‑OHT, detected by PI staining and flow cytometry (L) and quantified (M) (mean ± SD, n = 3, **P* < 0.001, two‑tailed unpaired Student's t‑test). (N) IC₅₀ values of H1299 cells with or without AKR1B10 knockout treated with 4‑OHT for 72 h, assessed by CCK‑8 assay. IC₅₀ values were calculated by nonlinear regression (mean ± SD, n = 3, two‑way ANOVA). (O) Body weight of xenograft‑bearing mice receiving DMSO or 4‑OHT treatment (mean ± SD, n = 5, ns, not significant, *P* > 0.05, two‑way ANOVA with Tukey's post‑hoc test). (P) Kaplan–Meier overall survival curves for TCGA lung cancer cohort stratified by AKR1B10 mRNA expression. Statistical significance was determined by the log‑rank test. (Q) Kaplan–Meier overall survival analysis of patients with chemotherapy‑resistant lung cancer stratified by AKR1B10 expression based on the auto‑selected optimal cutoff. Statistical significance was determined by the log‑rank test. (R) Pearson correlation of AKR1B10 and TUBB3 mRNA expression. Analysis was conducted via the GEPIA2 online tool. (S) Pearson correlation of AKR1B10 and RRM1 mRNA expression. Analysis was conducted via the GEPIA2 online tool. (T) Pearson correlation of AKR1B10 and TYMS mRNA expression. Analysis was conducted via the GEPIA2 online tool.

**Figure S8** **4-OHT induces noncanonical ferroptosis by targeting AKR1B10.** (A) H1299 cells were treated with 4‑OHT (0, 10, or 20 μM) for 48 h, and Western blot analysis was used to detect ACC1 expression. Vinculin served as a loading control. Representative blots from three independent experiments are shown. (B) Western blot analysis of ACC1 expression in H1299 stable cell lines with AKR1B10 knockout. Vinculin served as a loading control. Representative blots from three independent experiments are shown. (C) ACC1 mRNA levels in H1299 cells treated with 4-OHT (0, 10, or 20 μM) for 48 h. GAPDH served as the internal control. (mean ± SD, n = 3, ns, not significant, *P* > 0.05, one‑way ANOVA with Dunnett's post‑hoc test). (D) H1299 cells with or without AKR1B10 knockout (sgCtrl or sg‑AKR1B10) were treated with cycloheximide (CHX, 50 μg/mL) alone, or sgCtrl cells were treated with CHX plus 4‑OHT (20 μM) in the presence or absence of MG132 (10 μM) for the indicated time points. ACC1 protein levels were analyzed by Western blot analysis. Vinculin served as a loading control. Representative blots from three independent experiments are shown.

**Figure S9 NANA also induces noncanonical ferroptosis by targeting AKR1B10.** (A) Quantitative analysis of relative AKR1B10 protein levels derived from CETSA immunoblots. AKR1B10 band intensities were normalized to Vinculin (mean ± SD, n = 3, ****P* < 0.001, two‑tailed unpaired Student‘s t‑test). (B and C) A549 (B) and H1299 (C) cells were treated with Nanatinostat (NANA, 1.5 μM) with or without various cell death pathway inhibitors for 48 h. Cell viability was assessed by CCK-8 assays (mean ± SD, n = 3, ***P*  < 0.01;****P* < 0.001, one‑way ANOVA with Tukey‘s post‑hoc test). (D and E) Quantification of ACC1 protein levels in A549 (D) and H1299 (E) cells treated with NANA (0, 1, or 2 μM) for 48 h. Band intensities were normalized to Vinculin (mean ± SD, n = 3, **P* < 0.001, one‑way ANOVA with Dunnett's post‑hoc test). (F and G) Quantification of GPX4 protein levels in A549 (F) and H1299 (G) cells treated with NANA (0, 1, or 2 μM) for 48 h. Band intensities were normalized to Vinculin (mean ± SD, n = 3, **P* < 0.001, one‑way ANOVA with Dunnett's post‑hoc test).

**Figure S10** **4-OHT synergizes with RCSs‑generating chemotherapy drugs.** (A and B) Dose-response curves of H1299 cells treated with carboplatin (A) or paclitaxel (PTX) (B) for 72 h. Cell viability was assessed by CCK-8 assay. IC_50_ values were calculated by nonlinear regression (mean ± SD, n = 3, two-way ANOVA). (C and D) Synergy analysis of 4-OHT in combined with carboplatin (C) or paclitaxel (D) in H1299 cells for 72 h. Synergy scores were calculated using the zero‑interaction potency (ZIP) model.

(E) The Chou-Talalay combination index (CI) assay demonstrated that 4-OHT synergizes with carboplatin in A549 cells (mean CI = 0.627). (F) The Chou-Talalay combination index assay demonstrated that 4-OHT synergizes with paclitaxel (PTX) in A549 cells (mean CI = 0.525). (G) The Chou-Talalay combination index assay demonstrated that 4-OHT synergizes with carboplatin in H1299 cells (mean CI = 0.690).

(H) The Chou-Talalay combination index assay demonstrated that 4-OHT synergizes with paclitaxel (PTX) in H1299 cells (mean CI = 0.669). (I) The Chou-Talalay combination index assay demonstrated that 4-OHT synergizes with carboplatin in LLC cells (mean CI = 0.563). (J) The Chou-Talalay combination index assay demonstrated that 4-OHT synergizes with paclitaxel (PTX) in LLC cells (mean CI = 0.766).

**Supplemental Tables**

**Table S1.** The detailed information of 950 human endogenous metabolic compounds.

**Table S2.** The results of the untargeted metabolomics.

**Table S3.** The identified characteristic sequences by LiP-MS (4-OHT high-dose group VS Control group).

**Table S4.** The identified characteristic sequences by LiP-MS (4-OHT low-dose group VS Control group).

**Table S5.** The detailed pathological data of LUAD tissue microarray.

**Table S6.** The Top200 compounds identified by High-Throughput Virtual Screening using the Natural Product Library.

**Table S7.** The Top200 compounds identified by High-Throughput Virtual Screening using the Drug Repurposing Compound Library.

**Table S8.** The resources table.
